# Pitchers of *Nepenthes khasiana* express several digestive-enzyme encoding genes, harbor mostly fungi and probably evolved through changes in the expression of leaf polarity genes

**DOI:** 10.1186/s12870-020-02663-2

**Published:** 2020-11-17

**Authors:** Jeremy Dkhar, Yogendra Kumar Bhaskar, Andrew Lynn, Ashwani Pareek

**Affiliations:** 1 Stress Physiology and Molecular Biology Laboratory, School of Life Sciences, Jawaharlal Nehru University, New Delhi, 110067 India; 2 Agrotechnology Division, CSIR-Institute of Himalayan Bioresource Technology, Palampur, Himachal Pradesh 176061 India; 3 School of Computational and Integrative Sciences, Jawaharlal Nehru University, New Delhi, 110067 India

**Keywords:** *Nepenthes khasiana*, Leaf transcriptome, Pitcher development and evolution, Prey digestion, Plant defense

## Abstract

**Background:**

A structural phenomenon seen in certain lineages of angiosperms that has captivated many scholars including Charles Darwin is the evolution of plant carnivory. Evidently, these structural features collectively termed carnivorous syndrome, evolved to aid nutritional acquisition from attracted, captured and digested prey. We now understand why plant carnivory evolved but how carnivorous plants acquired these attributes remains a mystery. In an attempt to understand the evolution of *Nepenthes* pitcher and to shed more light on its role in prey digestion, we analyzed the transcriptome data of the highly specialized *Nepenthes khasiana* leaf comprising the leaf base lamina, tendril and the different parts/zones of the pitcher tube viz. digestive zone, waxy zone and lid.

**Results:**

In total, we generated around 262 million high-quality Illumina reads. Reads were pooled, normalized and de novo assembled to generate a reference transcriptome of about 412,224 transcripts. We then estimated transcript abundance along the *N. khasiana* leaf by mapping individual reads from each part/zone to the reference transcriptome. Correlation-based hierarchical clustering analysis of 27,208 commonly expressed genes indicated functional relationship and similar cellular processes underlying the development of the leaf base and the pitcher, thereby implying that the *Nepenthes* pitcher is indeed a modified leaf. From a list of 2386 differentially expressed genes (DEGs), we identified transcripts encoding key enzymes involved in prey digestion and protection against pathogen attack, some of which are expressed at high levels in the digestive zone. Interestingly, many of these enzyme-encoding genes are also expressed in the unopened *N. khasiana* pitcher. Transcripts showing homology to both bacteria and fungi were also detected; and in the digestive zone, fungi are more predominant as compared to bacteria. Taking cues from histology and scanning electron microscopy (SEM) photomicrographs, we found altered expressions of key regulatory genes involved in leaf development. Of particular interest, the expression of class III *HOMEODOMAIN-LEUCINE ZIPPER* (*HD-ZIPIII*) and *ARGONAUTE* (*AGO*) genes were upregulated in the tendril.

**Conclusions:**

Our findings suggest that *N. khasiana* pitchers employ a wide range of enzymes for prey digestion and plant defense, harbor microbes and probably evolved through altered expression of leaf polarity genes.

## Background

**Fig. 1:**
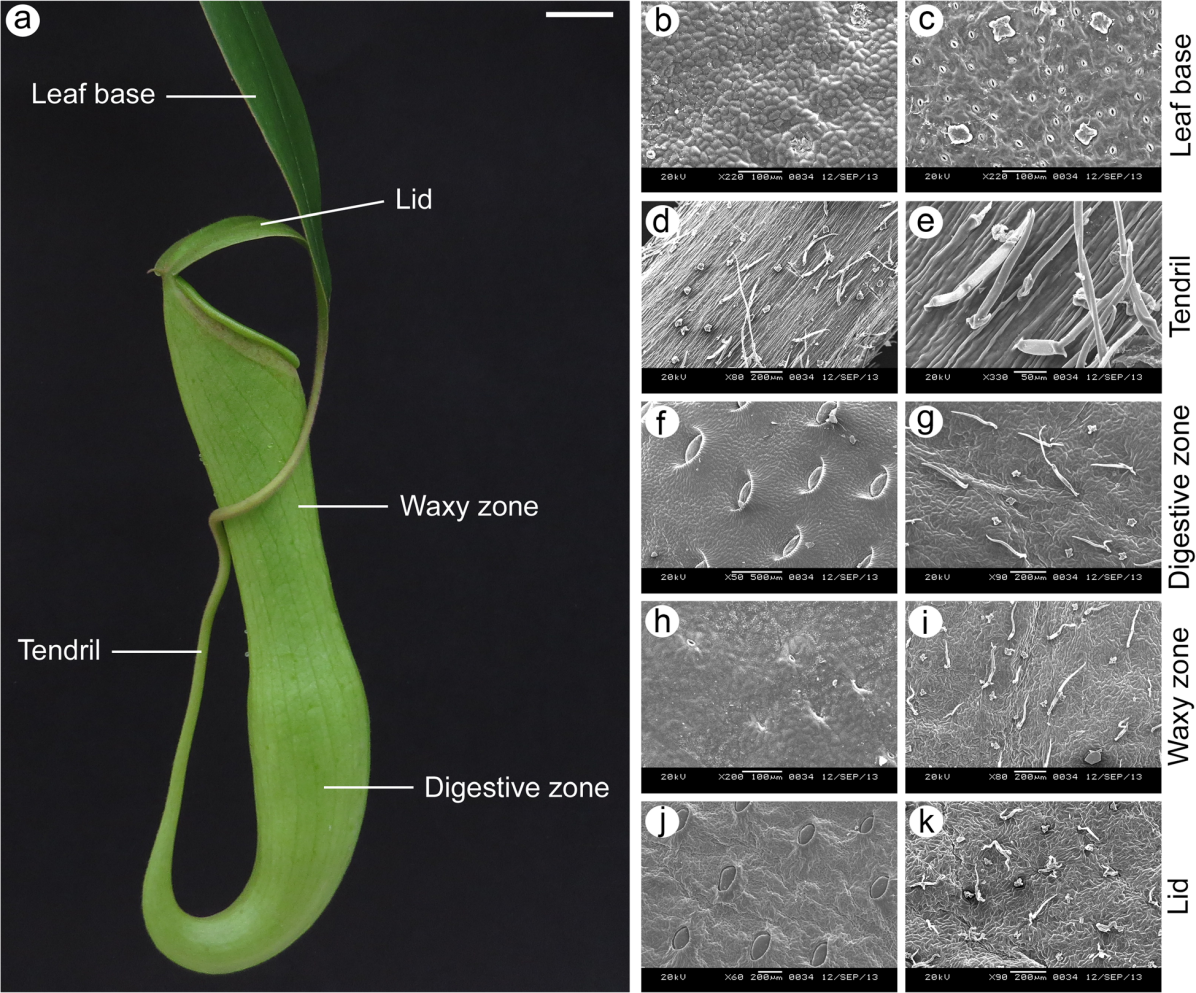
*Nepenthes khasiana* leaf. **a**, the five distinct parts/zones of the *N. khasiana* leaf comprising the leaf base, tendril, digestive zone, waxy zone and lid (bar = 1 cm). **b-k**, SEM photomicrographs of the leaf base (**b, c**), tendril (**d, e**), digestive zone (**f, g**), waxy zone (**h, i**) and lid (**j, k**). Barring tendril, SEM images on the left represent the adaxial surfaces while the ones on the right depict the abaxial surfaces. **e** is a close-up of the tendril shown in (**d**)

Carnivorous plants are remarkable botanical entities that are of considerable interest in the context of plant adaptation. These plants have evolved several times independently in five angiosperm lineages and are characterized by a set of features termed carnivorous syndrome [1, 2]. This syndrome is reflected mostly in the leaves to facilitate the attraction, capture and digestion of prey and the subsequent absorption of the dissolved nutrients to offset low nutrient availability in their natural habitat. Among these highly specialized leaves are the pitfall traps or ‘pitchers’ recognized in three families viz. Cephalotaceae, Nepenthaceae and Sarraceniaceae [3]. Members of the family Sarraceniaceae develop pitchers that function both in prey trapping and photosynthesis whereas *Nepenthes* and *Cephalotus* produce pitchers that can capture prey with little or no photosynthesis [4]. However, and unlike pitchers of *Sarracenia* and *Cephalotus*, the *Nepenthes* pitcher is attached at the base via a rigid slender structure called tendril to a flattened photosynthesizing leaf base lamina (Fig. 1). It is further divided into two anatomically and functionally distinct zones: a slippery waxy zone covering the upper inner part of the pitcher that function in prey trapping, and a basal digestive zone entrenched with enzyme secreting glands capable of absorbing the available nutrients (Fig. 1). Covering the mouth of the pitcher is a nectary gland-bearing lid that functions in prey attraction as well as in shielding rainwater from diluting the digestive juice [5]. This extraordinary attribute has fascinated scientists worldwide and research has progressed in the direction of understanding the mechanism of entrapment and digestion.

For elucidating the trapping mechanism of *Nepenthes* pitchers, studies have focused on the slippery waxy zone [6], the wettable peristome [7–9] and the viscoelastic digestive fluid [10, 11]. Once trapped inside the fluid-containing pitcher, insects begin to die and are digested through several hydrolytic enzymes. These enzymes include aspartic or cysteine proteinase, chitinase, ribonuclease, esterase, phosphatase, β-1,3-glucanase and β-D-xylosidase (Biteau et al. [12] and references therein). But the source of these enzymes remains a debatable question, to date. This situation arises from the contradictory results that showed either presence [13, 14] or absence [12, 15] of microbes in the digestive fluids of certain unopened *Nepenthes* pitchers. Whether *N. khasiana* pitchers harbor microbes are not yet known; however, we do know that it contains genes encoding chitinase enzymes [16] and it produces the antifungal secondary metabolite naphthoquinones [17]. Beyond this, no other report is available on pathogenesis-related or prey digestion genes in *N. khasiana*. Moreover, whether similar numbers of genes are expressed in both opened and unopened pitchers of *Nepenthes* remains to be explored.

To address these issues, we carried out a sequencing-based transcriptome profiling of the highly specialized *N. khasiana* leaf comprising the leaf base lamina, tendril and the different parts/zones of the pitcher tube viz. digestive zone, waxy zone and lid. Our analysis of the transcriptome data suggests that the *Nepenthes* pitcher is indeed a modified leaf. This is based on the observation that in comparison to the tendril, the leaf base lamina shares similar transcript expression patterns with the different parts/zones of the pitcher tube. We found that in the presence of captured prey or pathogenic microbes (open pitcher), almost all transcripts encoding key enzymes known to play a role in prey digestion and protection against pathogen attack are expressed in the *N. khasiana* pitcher. Unexpectedly, many of these enzyme-encoding genes are also expressed in the unopened pitcher; but in comparison to the open pitcher, the number of genes expressed is reduced. For instance, nepenthesin I and II are expressed in both the unopened and open pitchers whereas class IV chitinase is specifically expressed in the open pitcher. We also detected transcripts of microbial origin i.e. bacteria and fungi in all the five different parts/zones of the *N. khasiana* leaf; but in the digestive zone, these transcripts shared homology mostly to those of fungi. We also observed altered expressions of class III *HOMEODOMAIN-LEUCINE ZIPPER* (HD-ZIPIII) and *ARGONAUTE* (*AGO*) genes, thereby suggesting that genes specifying leaf polarity may play a key role in the development of the *Nepenthes* pitcher. Our findings suggest that *N. khasiana* pitchers employ a wide range of enzymes for prey digestion and plant defense, many of which are expressed prior to the opening of the lid, harbor mostly fungi in the digestive zone and probably evolved through altered expression of leaf polarity genes.

## Results

### Sequencing, de novo assembly and annotation

RNA sequencing of the five different parts/zones of the *N. khasiana* leaf resulted in a total of 262 million high-quality paired-end Illumina reads (Additional file 1: Table S1). Reads were combined into a single data set and assembled using the freely available software Trinity [18, 19] by applying the default settings to generate a reference de novo assembled transcriptome of the *N. khasiana* leaf. Redundant transcripts were removed from the Trinity generated assembly using cd_hit_est. The reference transcriptome contains 412,224 transcripts with a mean contig length and a maximum contig length of 0.695 kb and 23.743 kb, respectively. The N50 value is 1.356 kb. Figure S1 in Additional file 1 shows the length distribution of all assembled transcripts. Using BLASTX program [20], we then compared the assembled transcripts of length ≥ 200 bp with the NCBI non-redundant protein database and retained matches with E-value cut-off ≤10^− 5^ and similarity score ≥ 40%. We found 99,604 assembled transcripts possessed at least one significant hit against the NCBI non-redundant protein database. At least 1e^− 5^ confidence level was observed for around 60% of the transcripts, indicating high protein conservation (Additional file 1: Fig. S2a). About 79% of the transcripts possessed protein level similarity of more than 60% (Additional file 1: Fig. S2b). The predicted proteins from BLASTX were annotated against UniProt database. Out of 99,604 transcripts, 50,222 transcripts matched proteins available in the UniProt database. The organisms’ names corresponding to the top BLASTX hit of each transcript was extracted and plotted in Additional file 1: Fig. S3. *Beta vulgaris subsp. vulgaris* emerges as the top organism with 9292 matching transcripts. ‘Carbohydrate degradation’ (249), ‘amino-acid biosynthesis’ (199) and ‘protein modification’ (126) were among the most abundant metabolic pathways mapped (Additional file 1: Fig. S4). Figure S5 in Additional file 1 shows the top 10 GO terms identified in each category. Under the biological process category, ‘DNA integration’ is placed at the top while ‘integral component of membrane’ emerged as the top GO term under cellular component category. The molecular function category is represented at the top by ‘nucleic acid binding’. We have submitted the RNA-seq data from the two biological replicates generated in this study to NCBI Short Read Archive and it can be accessed under accession number SRP064181.

### Transcript abundance estimation and differentially expressed genes

**Fig. 2:**
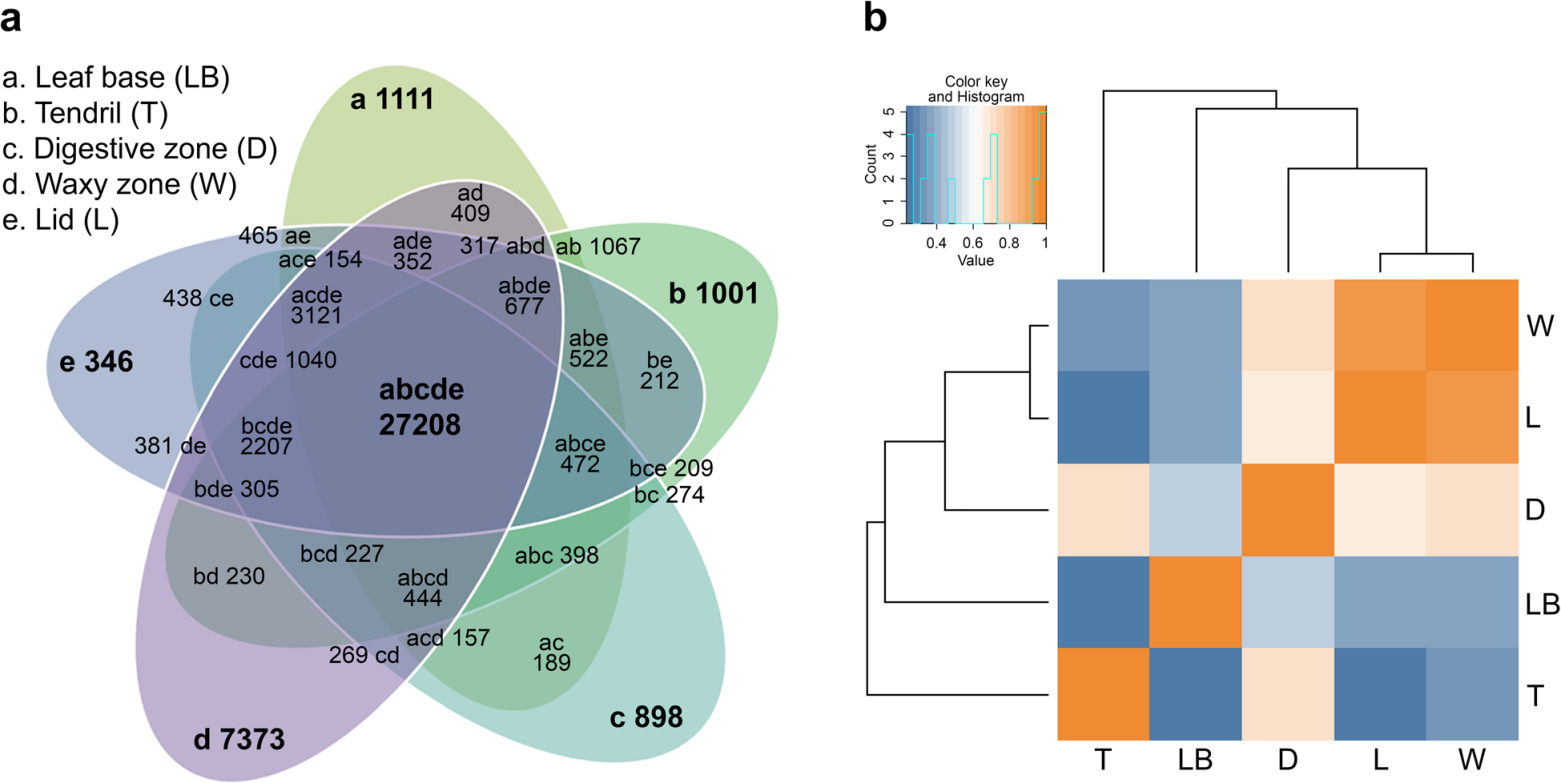
Estimation of transcript abundance and correlation-based hierarchical clustering analysis. **a**, Unique and shared transcripts in/among the five different parts/zones of the *N. khasiana* leaf. Numbers represent expressed transcripts. **b**, correlation-based hierarchical clustering of the five different samples based on the log_2_ FPKM values of 27,208 commonly expressed genes (red, positive correlation; blue, negative correlation). LB: leaf base; T: tendril; D: Digestive zone; W: waxy zone; L: lid

We estimated transcript abundance along the *N. khasiana* leaf by mapping individual reads from each part/zone to the reference transcriptome (length ≥ 200 bp) using Bowtie 2 [21]. About 95% of reads, on average, were properly aligned to the reference transcriptome. The alignment summary is provided in Additional file 1: Table S2. We then extracted unique and shared transcripts in and among all the different tissue parts/zones. We detected highest number of uniquely expressed transcripts in the waxy zone (7373), followed by leaf base (1111), tendril (1001), digestive zone (898) and lid (346) (Fig. 2a). In the waxy zone, 24 GO molecular function terms are enriched, of which 15 are over-represented and 9 are under-represented. ‘Protein dimerization activity’ and ‘oxidoreductase activity’ emerged at the top for under- and over-represented molecular functions, respectively (Additional file 1: Fig. S6). Two GO molecular function terms showed enrichment in the leaf base, of which ‘oxidoreductase activity’ is over-represented and ‘binding’ is under-represented. In the tendril, ‘RNA-directed DNA polymerase activity’ and ‘cysteine-type peptidase activity’ make up for the two over-represented GO molecular function terms. Four GO molecular function terms were enriched in the digestive zone, while no enrichment was detected for the lid (Additional file 1: Fig. S6). Surprisingly, the uniquely expressed genes contributing to the top molecular function term (‘structural constituent of cuticle’) in the digestive zone showed homology to insect cuticle proteins. All five parts/zones of the *N. khasiana* leaf shared a common set of 27,208 expressed transcripts (Fig. 2a). From the correlation analysis of the five different samples, the waxy zone and lid as well as the digestive zone showed high correlation among each other (Fig. 2b). At the same time, these distinct parts/zones of the pitcher tube displayed a relatively higher correlation with the leaf base than the tendril. This implies that the leaf base and the pitcher share similar transcripts expressions patterns.

**Fig. 3:**
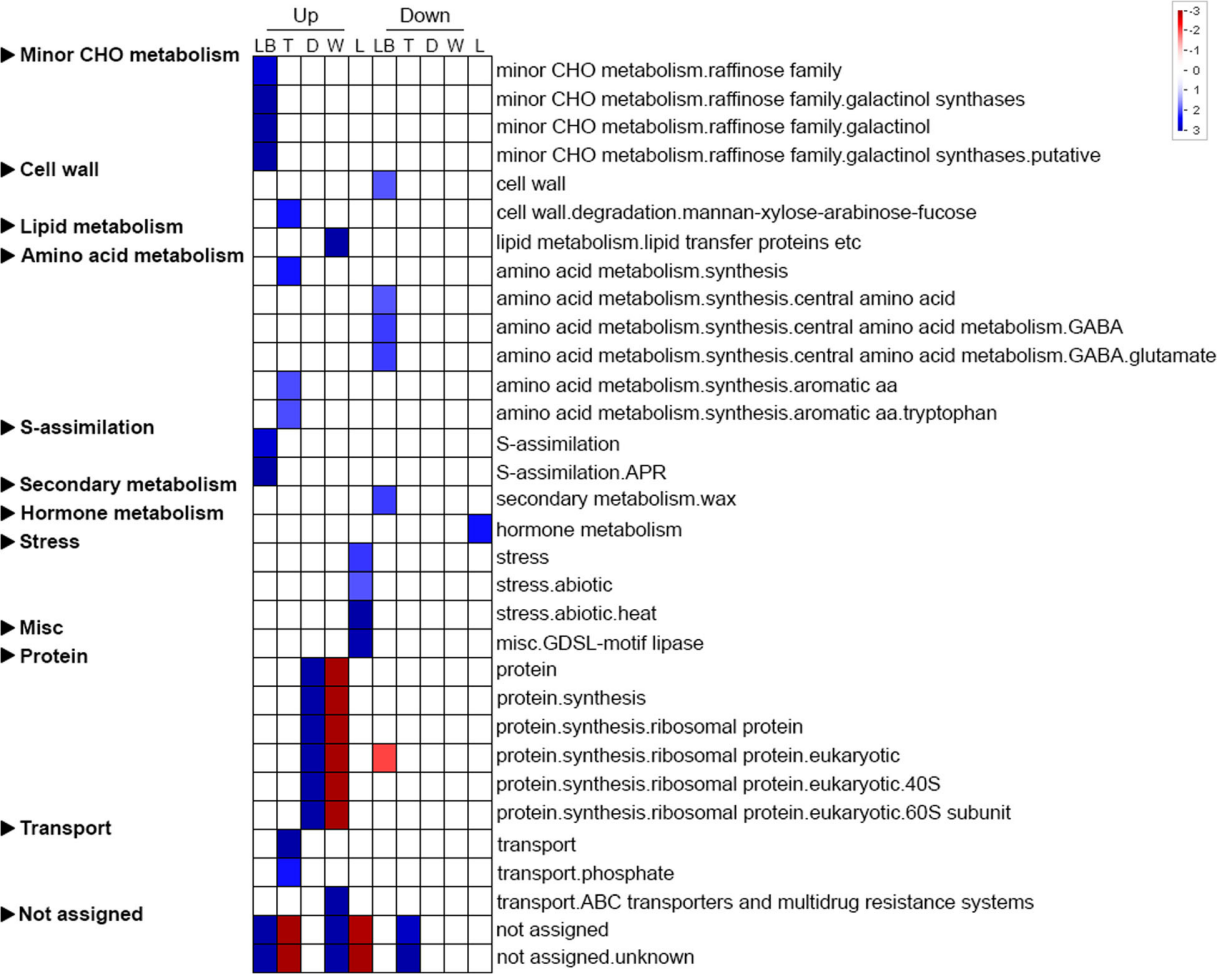
Overrepresentation analysis of up- and downregulated genes from the five parts/zones of *N. khasiana* leaf within functional gene classes defined by Mapman bins. Blue, up- or downregulated genes are significantly overrepresented; red, up- or downregulated genes are significantly underrepresented. LB: leaf base; T: tendril; D: Digestive zone; W: waxy zone; L: lid

We then used the DeSeq software [22] to generate the read counts and fragments per kilobase of transcript per million mapped reads (FPKM) values. Figure S7 in Additional file 1 shows a distribution of the FPKM values. On the basis of the applied criteria [*p*-value < 0.05], we identified 12,610 significantly DEGs along the *N. khasiana* leaf. Upon adjusting the *p*-value, the number of significantly DEGs reduces to 2386. The automated annotation software Mercator [23] was then used to generate a mapping file of the DEGs for overrepresentation and functional category enrichment analyses. The Mercator result shows that 56% of the data were assigned functions while no functions were assigned to the remaining 44% of the data (Additional file 1: Fig. S8). Overrepresentation analysis using Pageman [24] indicated that most enriched functions are specific to certain parts/zones of the *N. khasiana* leaf viz. minor CHO metabolism, cell wall, lipid metabolism, amino acid metabolism, S-assimilation, secondary metabolism, hormone metabolism, stress, misc. and transport (Fig. 3). Among the up-regulated genes, protein synthesis represents one of the enriched molecular functions overrepresented in the digestive zone of *N. khasiana* pitcher. It was earlier shown in the carnivorous plant *Dionaea muscipula* that de novo protein synthesis occurs simultaneously with the secretion of the digestive fluid, and part of the newly synthesized protein is also directly secreted into the fluid [25]. In light of this finding, our results suggest that de novo protein synthesis is also taking place in the pitcher of *N. khasiana*. Some genes were not assigned any functions and may represent those that are specific to *N. khasiana* (Fig. 3).

### K-means clustering and functional category enrichment analysis

**Fig. 4:**
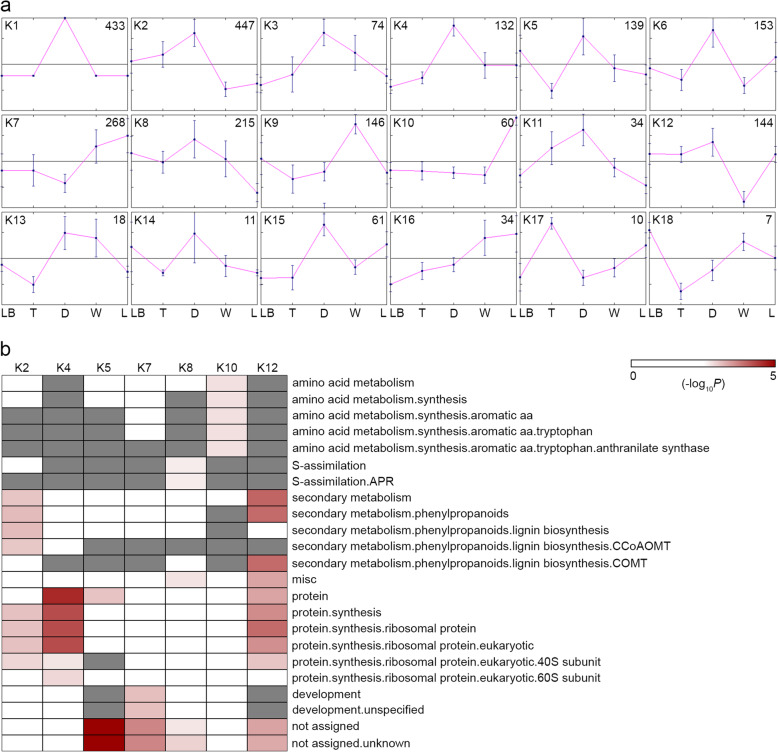
Clustering and functional category enrichment analyses of 2386 DEGs. **a**, 18 k-means clusters were identified along the five different parts/zones of the *N. khasiana* leaf, with each cluster showing different expression patterns (numbers denote the number of DEGs in each cluster; error bars denote mean ± SE). **b**, functional category enrichment (MapMan bins) among the 7 major clusters (No enrichment for remaining 11 clusters). Red, significant enrichment; white, non-significant; gray, not-detected

The significantly DEGs were then grouped according to the k-means clustering algorithm. Prior to k-means clustering, the number of clusters k was estimated using the Figures of Merit (FOM) application embedded in the MeV program [26]. The results show that the adjusted FOM decreases sharply and begins to level out after reaching 4 clusters (Additional file 1: Fig. S9). In addition to FOM, employing the gap statistic algorithm in R program resulted in 6 clusters (Additional file 1: Fig. S10). Therefore, the k-means clustering analysis was performed three times with each run generating 6 clusters using the K-means / K-medians Support Module (KMS) of the MeV program and applying the Kendall tau rank correlation. The final output consists of 18 consensus clusters in which all the member genes clustered together in at least 80% of the K-Means runs (Fig. 4a). Cluster 1–6, 8, 11, 12, 14, and 15 consisted of genes that showed relatively higher expression in the digestive zone whereas genes of cluster 9 are expressed at higher levels in the waxy zone. Cluster 10 and 17 are represented by genes that are highly expressed in the lid and tendril, respectively. Cluster 18 comprises of genes that are expressed at higher levels in the leaf base. Genes of cluster 13 are expressed at higher levels in both the digestive and the waxy zones whereas genes of cluster 7 and 16 showed high expression in both the waxy zone and lid.

To test for the enrichment of Mapman functional categories in each cluster, a Wilcoxon statistic followed by the Benjamini-Hochberg correction was performed. Seven out of the 18 clusters showed various enriched functions (Fig. 4b). But most of the functional categories are enriched in cluster 12. These include secondary metabolism, misc., and protein synthesis. The DEGs grouped in cluster 2 showed enrichment for secondary metabolism and protein synthesis. Protein synthesis is also enriched in cluster 4 whereas genes of cluster 5 showed enrichment for functions that are not assigned. Cluster 7 showed enrichment for development and unassigned functions. S-assimilation and amino acid metabolism are enriched in cluster 8 and 10, respectively. It was reported earlier that the concentration of aromatic amino acids increases during petal development in snapdragon, probably to attract potential pollinators [27]. Therefore, aromatic amino acid metabolism in the lid of the *N. khasiana* pitcher may be associated with the attraction of insect prey. The ‘not assigned’ function is also enriched in cluster 5, 8 and 12, and may contain genes that are specific to *N. khasiana* (Fig. 4b).

### Transcriptome profiling identifies genes involved in prey digestion and plant defense

**Fig. 5:**
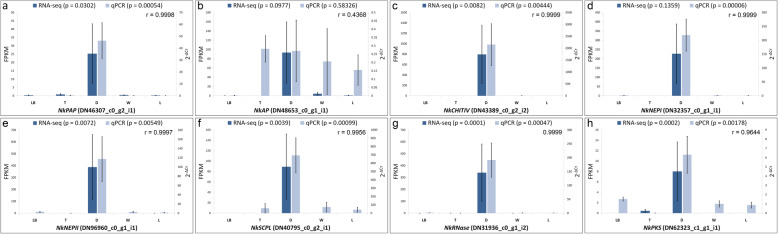
Graphical representation of the RNA-seq derived expression patterns of genes known to play a role in prey digestion and plant defense. Corresponding qPCR data for validation is also plotted (‘p’ denotes *p* value; *n* = 4; error bars indicate standard error). Pearson correlation (r) between the two data for each gene is also indicated. Expanded forms of each gene are represented in Additional file 1: Table S3. Codes within parentheses represent transcript IDs. LB: leaf base; T: tendril; D: digestive zone; W: waxy zone; L: lid

Table 1 shows a list of transcripts sharing homology to enzyme-encoding genes known to play a role in prey digestion and plant defense. A complete list of all the enzymes detected in the different parts/zones of the *N. khasiana* leaf along with their descriptions, functions, transcript IDs and abundances can be found in Additional file 2: Table S4. Among them, aspartic proteinases nepenthesin I and nepenthesin II (DN32357_c0_g1_i1, DN96960_c0_g1_i1), class IV chitinase (DN167792_c0_g1_i1, DN43389_c0_g2_i1, DN43389_c0_g2_i2), C-terminal peptidase (DN61492_c2_g2_i1, DN61492_c2_g2_i2), defensin (DN3077_c0_g2_i1), GDSL esterase/lipase (DN43304_c0_g1_i1, DN43304_c0_g2_i1), peroxidase (DN42192_c0_g1_i1, DN42192_c0_g1_i2), phosphatase (DN6769_c0_g2_i1), and serine carboxypeptidase (DN40795_c0_g2_i1, DN40795_c0_g2_i2) showed high levels of expression in the digestive zone with low or lack of expressions in the other parts/zones of the *N. khasiana* leaf (Table 1). Transcripts encoding type III polyketide synthase also showed high expression in the digestive zone. In addition, some transcripts encoding enzymes such as acid phosphatase (DN46307_c0_g2_i1, DN46369_c0_g1_i1 and DN46369_c0_g1_i4), acidic endochitinase (DN61615_c0_g1_i1 and DN61615_c0_g1_i2), C-terminal peptidase (DN61492_c2_g1_i9, DN61492_c2_g1_i2), glucanase (DN60686_c0_g1_i1), serine carboxypeptidase (DN55176_c1_g1_i1), S-like RNase (DN31936_c0_g1_i1, DN31936_c0_g1_i2) and thaumatin-like proteins (DN58459_c0_g2_i6, DN60173_c0_g2_i12, DN60173_c0_g2_i2) are expressed throughout the *N. khasiana* leaf with elevated levels in the digestive zone. A single transcript (DN14283_c0_g1_i1) which encodes a lipid transfer protein is also expressed throughout the *N. khasiana* leaf with increased expression in the pitcher tube. Validation of the RNA-seq data using real time qPCR analysis indicated a strong correlation between the two data (Fig. 5).

**Table 1:** List of enzymes detected in the different parts/zones of *N. khasiana* leaf with their corresponding transcript IDs, BLAST results and transcript abundance (FPKM) in the different parts/zones of the *N. khasiana* leaf. These enzymes were earlier detected/ isolated from *Nepenthes* pitcher fluids and known to play a role in prey digestion and protection against pathogen attack. A complete and more detailed list of all the enzymes detected in the *N. khasiana* leaf can be found in Additional file 2: Table S4

Sl. No.	Enzyme	Transcript ID	BLAST	Transcript abundance (FPKM)^a^					*P* value
				Leaf base	Tendril	Digestive zone	Waxy zone	Lid	
1	Acid phosphatase	DN46307_c0_g2_i1	PREDICTED: probable purple acid phosphatase 20 [*Gossypium hirsutum*]	0.31 ± 0.04	0.80 ± 0.05	25.32 ± 13.57	0.40 ± 0.17	0.20 ± 0.16	0.0302
		DN46369_c0_g1_i1	PREDICTED: probable purple acid phosphatase 20 [*Eucalyptus grandis*]	2.16 ± 1.44	1.37 ± 0.16	20.44 ± 0.19	0.74 ± 0.15	0.22 ± 0.08	0.0001
		DN46369_c0_g1_i4	PREDICTED: probable purple acid phosphatase 20 [*Eucalyptus grandis*]	2.42 ± 0.70	1.53 ± 0.24	23.33 ± 4.14	0.79 ± 0.31	0.12 ± 0.04	0.0002
2	Aspartic proteinase	DN32357_c0_g1_i1	RecName: Full = Aspartic proteinase nepenthesin-1; AltName: Full = Nepenthesin-I; Flags: Precursor	0.10 ± 0.08	0.00 ± 0.00	227.32 ± 188.36	0.00 ± 0.00	0.02 ± 0.03	0.1359
		DN46672_c0_g1_i1	Nepenthesin-2-like protein [*Nepenthes mirabilis*]	0.00 ± 0.00	0.02 ± 0.02	29.68 ± 18.62	0.00 ± 0.00	0.00 ± 0.00	0.0521
		DN48653_c0_g1_i1	Aspartic protease [*Nepenthes alata*]	0.12 ± 0.05	0.00 ± 0.00	93.48 ± 69.04	4.38 ± 0.78	0.83 ± 0.75	0.0977
		DN96960_c0_g1_i1	Nepenthesin II [*Nepenthes ovata*]	0.00 ± 0.00	0.00 ± 0.00	387.83 ± 150.84	0.07 ± 0.10	0.13 ± 0.19	0.0072
3	Chitinase	DN167792_c0_g1_i1	Class IV chitinase [*Nepenthes alata*]	0.33 ± 0.25	0.09 ± 0.03	569.95 ± 244.98	0.00 ± 0.00	0.00 ± 0.00	0.0112
		DN168202_c0_g1_i1	Basic endochitinase A [*Ananas comosus*]	0.00 ± 0.00	0.00 ± 0.00	21.45 ± 23.10	2.94 ± 1.74	0.46 ± 0.49	0.3042
		DN43389_c0_g2_i1	Class IV chitinase [*Nepenthes alata*]	0.08 ± 0.12	0.02 ± 0.03	426.64 ± 203.61	0.00 ± 0.00	0.00 ± 0.00	0.0175
		DN43389_c0_g2_i2	Class IV chitinase [*Nepenthes alata*]	0.19 ± 0.26	0.02 ± 0.03	795.95 ± 318.67	0.00 ± 0.00	0.03 ± 0.04	0.0082
		DN61615_c0_g1_i1	Acidic endochitinase [*Nepenthes singalana*]	129.27 ± 72.25	43.13 ± 9.23	1472.88 ± 71.20	41.40 ± 13.71	42.40 ± 2.44	0.0001
		DN61615_c0_g1_i2	Acidic endochitinase [*Nepenthes singalana*]	97.15 ± 117.68	29.70 ± 28.23	878.25 ± 912.14	29.37 ± 30.72	24.26 ± 23.22	0.2960
4	C-terminal peptidase	DN61492_c2_g1_i14	C-terminal peptidase [*Nepenthes alata*]	5.21 ± 1.74	4.03 ± 5.33	92.21 ± 15.31	0.23 ± 0.28	0.00 ± 0.00	0.0002
		DN61492_c2_g1_i9	C-terminal peptidase [*Nepenthes alata*]	10.72 ± 0.67	0.77 ± 0.01	156.87 ± 121.48	0.19 ± 0.27	0.04 ± 0.06	0.1155
		DN61492_c2_g2_i1	C-terminal peptidase [*Nepenthes alata*]	0.00 ± 0.00	0.02 ± 0.02	147.31 ± 2.44	0.00 ± 0.00	0.00 ± 0.00	0.0001
		DN61492_c2_g2_i2	C-terminal peptidase [*Nepenthes alata*]	0.02 ± 0.03	0.03 ± 0.01	237.00 ± 124.39	0.00 ± 0.00	0.00 ± 0.00	0.0259
		DN61492_c2_g1_i2	C-terminal peptidase [*Nepenthes alata*]	12.10 ± 8.00	4.19 ± 5.09	66.36 ± 21.25	0.24 ± 0.26	0.01 ± 0.02	0.0056
5	Defensin	DN3077_c0_g2_i1	PREDICTED: defensin Ec-AMP-D1-like [*Ziziphus jujuba*]	0.06 ± 0.09	0.00 ± 0.00	116.53 ± 8.96	0.00 ± 0.00	0.00 ± 0.00	0.0001
6	Esterase/lipase	DN43304_c0_g1_i1	GDSL esterase/lipase 7-like	0.00 ± 0.00	0.00 ± 0.00	7.12 ± 6.45	0.00 ± 0.00	0.00 ± 0.00	0.1777
		DN43304_c0_g2_i1	GDSL esterase/lipase 7-like	0.00 ± 0.00	0.00 ± 0.00	9.43 ± 9.72	0.00 ± 0.00	0.00 ± 0.00	0.2517
7	Glucanase	DN60686_c0_g1_i1	Glucanase [*Nepenthes khasiana*]	1.50 ± 2.13	28.92 ± 38.04	218.24 ± 254.42	2.80 ± 3.53	1.64 ± 2.16	0.3692
		DN24941_c0_g1_i3	Beta-1,3-glucanase [*Nepenthes alata*]	0.68 ± 0.96	46.74 ± 66.10	15.36 ± 21.72	0.38 ± 0.54	0.36 ± 0.51	0.5577
8	Lipid transfer protein	DN14283_c0_g1_i1	Lipid transfer protein 1b, partial [*Ipomoea batatas*]	0.57 ± 0.08	0.18 ± 0.03	498.38 ± 55.17	0.10 ± 0.15	0.10 ± 0.14	0.0001
9	Peroxidase	DN61358_c1_g2_i4	Putative peroxidase [*Nepenthes alata*]	5.13 ± 1.74	21.76 ± 13.13	5.27 ± 0.21	0.71 ± 0.40	1.44 ± 0.89	0.0744
		DN42192_c0_g1_i1	Putative peroxidase 27 [*Nepenthes mirabilis*]	0.00 ± 0.00	0.00 ± 0.00	19.64 ± 20.31	0.09 ± 0.13	0.00 ± 0.00	0.2547
		DN42192_c0_g1_i2	Putative peroxidase 27 [*Nepenthes mirabilis*]	0.00 ± 0.00	0.00 ± 0.00	87.53 ± 71.76	0.09 ± 0.13	0.13 ± 0.19	0.1317
10	Polyketide synthase	DN62323_c0_g1_i1	Type III polyketide synthase [*Drosophyllum lusitanicum*]	12.30 ± 12.19	11.93 ± 1.17	51.86 ± 3.84	1.47 ± 0.16	1.47 ± 1.04	0.0015
		DN62323_c0_g2_i1	Type III polyketide synthase [*Drosophyllum lusitanicum*]	15.71 ± 7.35	22.25 ± 13.43	98.14 ± 61.61	2.30 ± 1.33	2.71 ± 0.71	0.0814
		DN62323_c1_g1_i1	Type III polyketide synthase [*Drosophyllum lusitanicum*]	0.00 ± 0.00	0.44 ± 0.08	8.02 ± 1.50	0.00 ± 0.00	0.00 ± 0.00	0.0002
		DN62323_c2_g2_i1	Type III polyketide synthase [*Drosophyllum lusitanicum*]	2.06 ± 0.41	7.79 ± 4.81	36.59 ± 13.35	1.36 ± 0.07	0.43 ± 0.02	0.0095
		DN62323_c2_g2_i2	Type III polyketide synthase [*Drosophyllum lusitanicum*]	1.36 ± 1.40	4.61 ± 0.32	23.77 ± 4.78	0.95 ± 0.51	0.56 ± 0.16	0.0006
		DN62323_c2_g2_i6	Type III polyketide synthase [*Drosophyllum lusitanicum*]	12.95 ± 11.27	14.24 ± 2.11	56.92 ± 3.31	1.69 ± 0.46	1.62 ± 0.83	0.0007
		DN62323_c2_g2_i7	Type III polyketide synthase [*Drosophyllum lusitanicum*]	17.67 ± 4.59	28.56 ± 22.35	124.77 ± 99.27	2.81 ± 2.05	3.21 ± 1.42	0.1695
11	Phosphatase	DN6769_c0_g2_i1	Putative nucleotide pyrophosphatase/phosphodiesterase [*Nepenthes mirabilis*]	0.00 ± 0.00	0.00 ± 0.00	21.94 ± 18.02	0.00 ± 0.00	0.00 ± 0.00	0.1322
12	Ribonuclease	DN31936_c0_g1_i1	S-like ribonuclease [*Nepenthes bicalcarata*]	0.08 ± 0.11	0.29 ± 0.07	241.43 ± 183.27	0.13 ± 0.13	0.07 ± 0.10	0.1026
		DN31936_c0_g1_i2	S-like ribonuclease [*Nepenthes bicalcarata*]	0.15 ± 0.21	0.27 ± 0.09	340.78 ± 42.77	0.13 ± 0.13	0.09 ± 0.07	0.0001
13	Serine Carboxypeptidase	DN40795_c0_g2_i1	Putative serine carboxypeptidase type 3 [*Nepenthes mirabilis*]	0.00 ± 0.00	0.01 ± 0.02	89.08 ± 30.19	0.00 ± 0.00	0.00 ± 0.00	0.0039
		DN40795_c0_g2_i2	Putative serine carboxypeptidase type 3 [*Nepenthes mirabilis*]	0.02 ± 0.02	0.00 ± 0.00	65.12 ± 26.85	0.00 ± 0.00	0.00 ± 0.00	0.0093
		DN55176_c1_g1_i1	Serine carboxypeptidase II-3-like [*Sesamum indicum*]	0.09 ± 0.08	0.47 ± 0.03	28.15 ± 22.32	0.06 ± 0.03	0.06 ± 0.09	0.1203
14	Thaumatin-like protein	DN58459_c0_g2_i6	Pathogenesis-related protein [*Tamarix hispida*]	73.28 ± 41.43	55.69 ± 0.40	290.93 ± 160.73	14.97 ± 1.84	17.07 ± 6.08	0.0583
		DN60173_c0_g2_i12	Thaumatin-like protein [*Nepenthes* × *henryana*]	60.85 ± 6.96	55.96 ± 27.68	1925.58 ± 1622.94	734.79 ± 774.33	15.30 ± 18.35	0.2207
		DN60173_c0_g2_i2	Thaumatin-like protein [*Nepenthes* × *henryana*]	81.35 ± 49.79	160.64 ± 189.76	248.98 ± 337.33	113.83 ± 150.01	5.77 ± 1.11	0.7580

^a^FPKM values represent mean ± SD

### Genes known to play a role in prey digestion and plant defense are also expressed in un-opened pitchers of *N. khasiana*

**Fig. 6:**
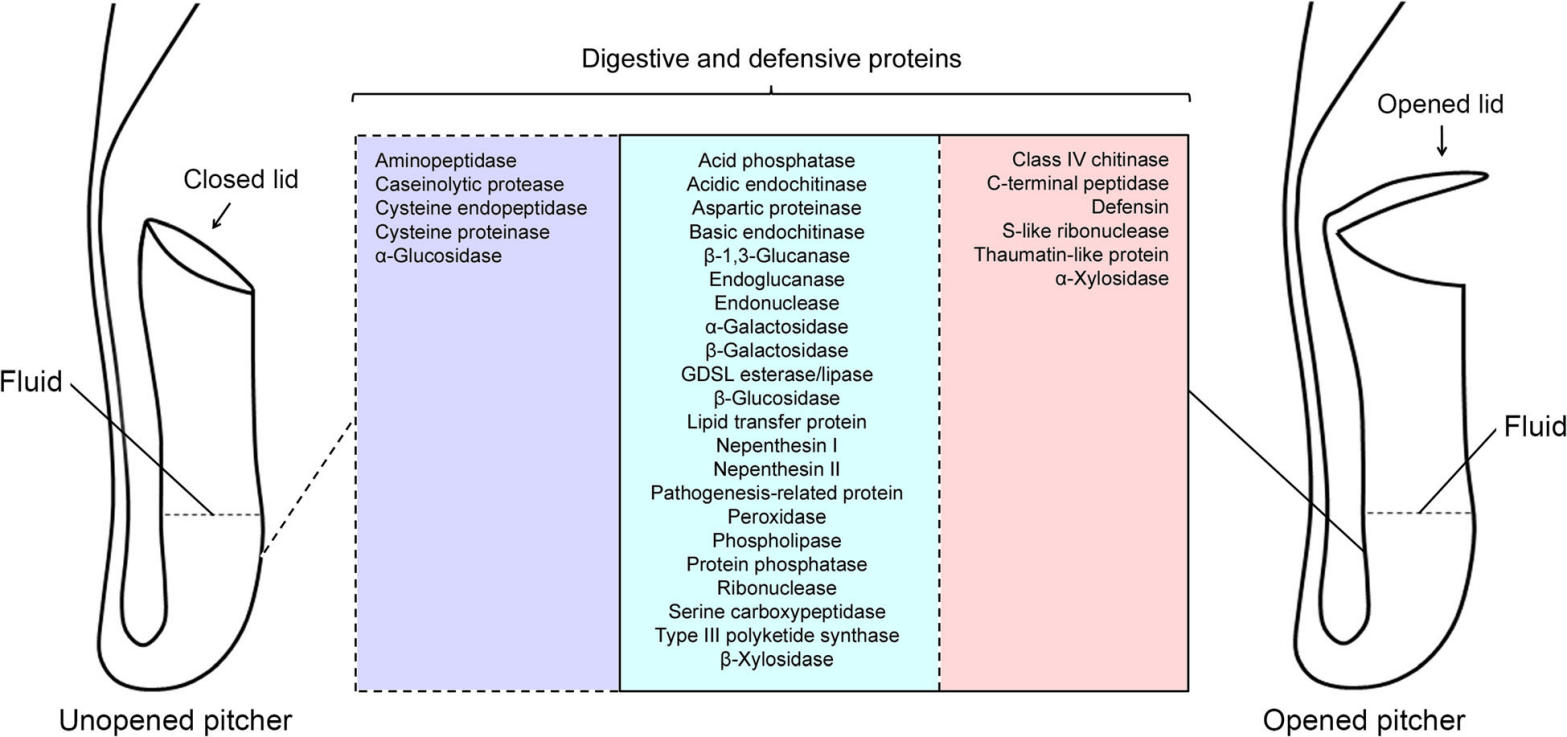
Diagrammatic representation of the digestive and defensive proteins expressed in the unopened (left side) and opened (right side) pitchers of *N. khasiana*. Proteins specific to the unopened pitcher are given in dashed line box (left side) whereas those that are exclusively expressed in the opened pitcher are given in solid line box (right side). Commonly expressed proteins are listed in the centre

A question arises as to whether similar kinds of digestive enzyme-encoding genes are also expressed in unopened *N. khasiana* pitchers, in which the lid is still attached to the pitcher tube (Additional file 1: Fig. S11). To address this question, we examined the transcriptome profile of an unopened pitcher generated independently of this study [28]. The results show that most transcripts sharing homology to key enzymes involved in prey digestion and plant defense are also expressed in unopened *N. khasiana* pitchers (Additional file 3: Table S5). Commonly expressed between the opened and unopened pitchers include genes that encode acid phosphatase, nepenthesin I and II, GDSL esterase/lipase, peroxidase, type III polyketide synthase, pathogenesis-related protein and several others whereas genes that encode for class IV chitinase, C-terminal peptidase, defensin, S-like ribonuclease, thaumatin-like protein and α-xylosidase are specifically expressed in the opened pitchers (Fig. 6). Thus, the presence of captured insects or pathogenic microbes, as a result of the opening of the lid, triggers the expression of more number of genes encoding other key digestive enzymes.

### Transcriptome data suggests the presence of microbial transcripts

Our RNA-seq results indicated the presence of transcripts showing homology to genes of microbial origin i.e. bacteria and fungi (Additional file 4: Table S6 and Additional file 5: Table S7). Transcripts of bacterial origin were mostly detected in the waxy zone, although a few were also identified in the digestive zone (Additional file 1: Fig. S12). In terms of the number of transcripts detected, the most abundant bacteria include *Chryseobacterium* (50), *Microbacterium* (34), *Micrococcus* (26), *Staphylococcus* (12), and *Streptococcus* (10). Although a number of transcripts that correspond to enzymes having proteolytic and nucleolytic activities viz. restriction endonuclease (DN49567_c0_g1_i1), LD-carboxypeptidase (DN53339_c0_g1_i2), M23 family peptidase (DN18977_c0_g1_i1), excinuclease ABC subunit B (DN55294_c1_g1_i1), and HNH endonuclease (DN19920_c0_g3_i1) were identified, these were detected on the waxy zone rather than the digestive zone.

We also detected transcripts of fungal origin, most of which are confined to the different parts/zones of the pitcher tube while some occur throughout the highly specialized *N. khasiana* leaf (Additional file 5: Table S7). Our data further suggest that fungi predominantly occur in the digestive zone as compared to bacteria (Additional file 1: Fig. S12). In cases where fungal transcripts were detected in the digestive zone, some correspond to genes encoding enzymes involved in protein degradation and hydrolysis of organic phosphates. These include acid phosphatase (DN225864_c0_g1_i1), acid protease (DN47168_c0_g1_i1, DN47168_c0_g2_i1), and peptidase C1B (DN94491_c0_g1_i1), all of which belonged to *Metschnikowia bicuspidata*, a fungal parasite. Interestingly, we identified beetle-borne fungi which include *Grosmannia clavigera*, *Lodderomyces elongisporus*, *Ophiostoma piceae* and *Spathaspora passalidarum*. Besides, an acidophilic filamentous fungus *Acidomyces richmondensis* and an ant-associated fungus *Phialophora attae* were also identified. Based on their transcript numbers, most dominant fungi include *Candida* (141), *Clavispora lusitaniae* (60), *Grosmannia clavigera* (12), *Kwoniella* (18), *Metschnikowia* (167), *Mycosphaerella eumusae* (11), *Ophiostoma piceae* (15) and *Sporothrix* (64).

Transcripts of microbial origin were also detected in the unopened pitchers of *N. khasiana* (Additional file 6: Table S8 and Additional file 7: Table S9). Although the number of bacteria and fungi detected is relatively small, both opened and unopened pitchers do share common transcripts of microbial origin. These transcripts belong to the fungal genera *Aspergillus* and *Talaromyces* and the bacterial genera *Aeromonas*, *Burkholderia*, *Pantoea* and *Pseudomonas*.

### Ultrastructure and anatomy of the highly specialized *N. khasiana* leaf

**Fig. 7:**
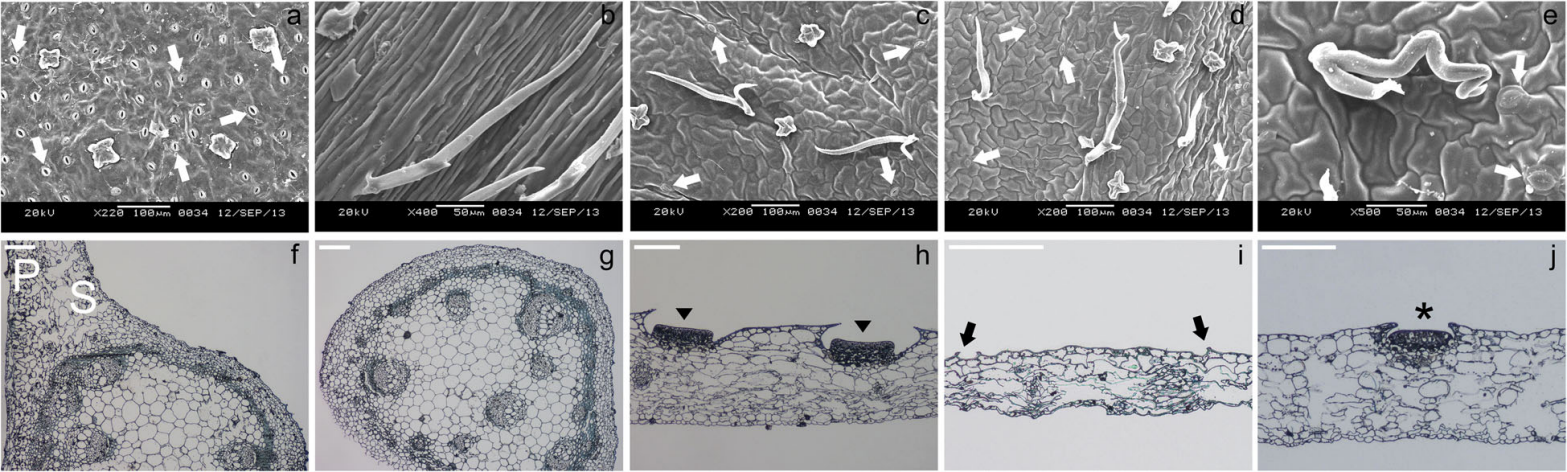
Stomatal density and distribution as well as leaf polarity in *N. khasiana* leaf. **a-e**, SEM photomicrographs of the abaxial surfaces of the different parts/zones of the *N. khasiana* leaf (stomata are indicated by white arrow). **f-j**, light micrographs cross sections of the five different parts/zones of the *N. khasiana* leaf (P: palisade parenchyma; S: spongy parenchyma; arrow head denotes digestive glands; black arrow shows the lunate cells; nectary gland is indicated by an asterisk). **a, f** - leaf base; **b, g** - tendril; **c, h** - digestive zone; **d, i** - waxy zone; **e, j** - lid. **f-j**, bar = 50 μm

SEM photomicrographs showed that the arrangement of stomata in the abaxial surface of the leaf base of *N. khasiana* does not follow the one-cell spacing rule as seen in the model plant *Arabidopsis thaliana* [29]. But the leaf base possesses more number of stomata as compared to the abaxial surfaces of the different parts/zones of the pitcher tube (Fig. 7a-e). On the other hand, the tendril completely lacks stomata (Fig. 7b). These observations find additional support from nail polish epidermal imprints, in which the leaf base recorded a stomatal index of 11.19 ± 0.96% while the digestive zone, waxy zone and lid recorded 0.97 ± 0.31%, 1.26 ± 0.33%, and 1.36 ± 0.42% stomatal indexes, respectively (Additional file 1: Fig. S13). Cross sections along the *N. khasiana* leaf showed that the adaxial/abaxial polarity is maintained in the leaf base but becomes indistinct in the different parts/zones comprising the pitcher tube (Fig. 7f-j).

### Expression patterns of key regulatory genes involved in stomatal development and leaf polarity specification

**Fig. 8:**
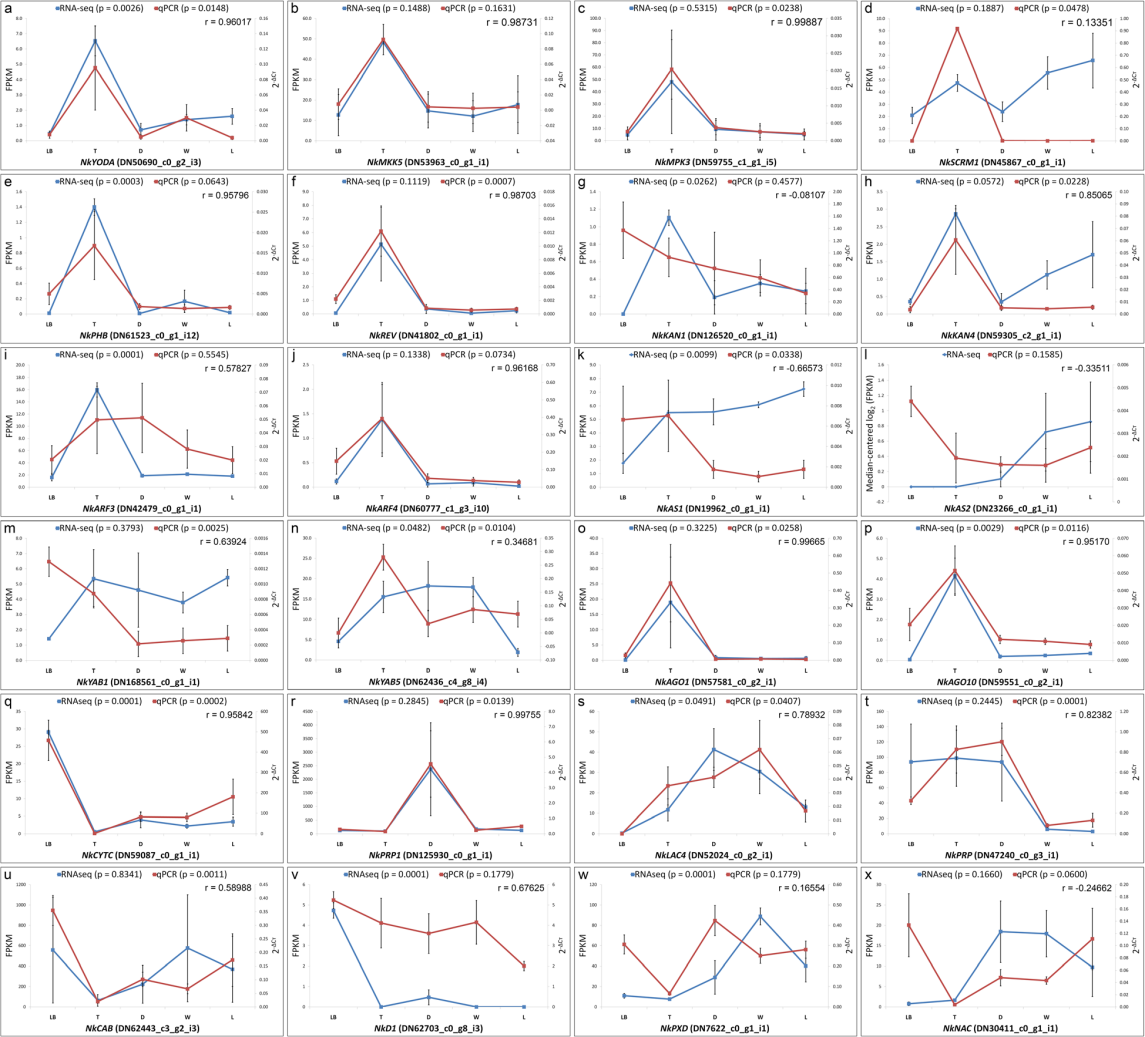
Graphical representation of the RNA-seq derived expression patterns of genes known to play a role in stomatal density and distribution (**a-d**) and leaf polarity specification (**e-p**) (blue line; refer Note S1 in Additional file 1). Corresponding qPCR data for validation is also plotted (red line; ‘p’ denotes *p* value; *n* = 4; error bars indicate standard error). **q-x** represent both RNA-seq and qPCR data of randomly selected genes for validation. Pearson correlation (r) between the two data for each gene is also indicated. LB: leaf base; T: tendril; D: Digestive zone; W: waxy zone; L: lid. The expanded form of each gene is represented in Additional file 1: Table S3

Taking cues from histology and SEM photomicrographs of the five distinct parts/zones of the *N. khasiana* leaf, we examined the expression pattern of genes involved in stomatal development and leaf polarity specification. Since the investigated samples represent mature tissues, we sought to determine the expression pattern of genes acting at the late stage of stomatal development. Based on the information available for the model plant Arabidopsis (see Additional file 1: Note S1) and assuming that similar genes control stomata formation in *N. khasiana*, we predicted that *NkYDA* and its downstream signaling components (*NkMKK4/NkMKK5* and *NkMPK3/NkMPK6*) would be downregulated in the leaf base and relatively lesser in the pitcher but upregulated in the tendril of *N. khasiana*. We also determined the expression of *NkSCRM*, anticipating an increased expression in the tendril considering the fact that loss-of-function *scrm* Arabidopsis mutants displayed increased stomatal densities [30]. Our results show that almost all genes behaved as predicted (Fig. 8a-d). In *N. khasiana*, the adaxial/abaxial polarity in the leaf base is maintained but in the different parts/zones comprising the pitcher tube, polarity is lost (Fig. 7f-j; also see Pavlovič et al. [4]). Because of this loss in polarity specification, we sought to determine the expression pattern of leaf polarity genes along the highly specialized *N. khasiana* leaf. Members of the class III *HOMEODOMAIN-LEUCINE ZIPPER (HD-ZIPIII)* gene family such as *PHABULOSA (PHB)*, *PHAVOLUTA (PHV)* and *REVOLUTA (REV)*, the *KANADI (KAN)* gene family encoding nuclear-localized GARP-domain transcription factors, the *AUXIN RESPONSE FACTOR* (*ARF*) gene family, the carboxy-terminal PAZ and PIWI domain containing *ARGONAUTE (AGO)* genes and the MYB domain *ASYMMETRIC LEAVES (AS)* 1 and LOB domain *AS2* transcription factors specify adaxial/abaxial leaf polarity in Arabidopsis. We found that the expression of transcripts homologous to Arabidopsis class III *HD-ZIP* (*NkPHB* & *NkREV*) and *ARGONAUTE* (*NkAGO1* & *NkAGO10*) genes were upregulated in the tendril (Fig. 8e, f, o, p). qPCR further validates the RNA-seq results (Fig. 8e, f, o, p). Figures 8q-x represent qPCR-based validation of additional RNA-seq data of randomly selected genes.

## Discussion

### Pitchers of *N. khasiana* employ a wide range of enzymes for prey digestion

Our analysis of the transcriptome data shows that *N. khasiana* pitchers employ a wide range of enzymes for prey digestion (Table 1). Among them, chitinase is the only enzyme that has previously been isolated and characterized in *N. khasiana* [16]. Using degenerate PCR, Eilenberg et al. [16] identified four class I chitinase genes in *N. khasiana* (NkChit1b-1 and 2, NkChit2b-1 and 2). They found that both chitinases are expressed in the pitcher (tissue) in response to the addition of chitin into closed *N. khasiana* pitcher traps to mimic captured prey. However, NkChit2b expression is also detected prior to chitin induction implying that this enzyme may represent a constitutively expressed housekeeping chitinase. Our results corroborated their findings whereby two (DN61615_c0_g1_i1, DN61615_c0_g1_i2) out of six detected transcripts sharing homology to acidic endochitinases showed expression throughout the different parts/zones of the *N. khasiana* leaf. The expression of one transcript (DN168202_c0_g1_i1) is restricted to the pitcher tube while the remaining three transcripts (DN167792_c0_g1_i1, DN43389_c0_g2_i1, DN43389_c0_g2_i2), which corresponds to class IV chitinase, are expressed at high levels in the digestive zone. The latter transcripts match the one identified by Hatano & Hamada in *N. alata*, and their protein products are most likely secreted into the pitcher fluid of *N. khasiana* to aid prey digestion and protection against pathogen attack.

Our results further indicated that transcripts (DN32357_c0_g1_i1, DN96960_c0_g1_i1) with sequence homology to nepenthesin I and II are expressed at high levels in the digestive zone of *N. khasiana*. Nepenthesin belongs to a different class of acid proteinases with specificity to aspartic acid residues [31]. Their relationship (i.e. nepenthesin I and II) with other aspartic proteinases (APs) is unclear; hence are regarded as a novel subfamily of APs with the distinction of being secreted into the extracellular space (digestive fluid), unlike other orthologous APs [31]. Likewise, and based on the RNA-seq derived expression patterns, the putative nepenthesin I and II of *N. khasiana* are probably secreted into the digestive fluid to help in protein degradation. On the contrary, the expressions of four APs homologs isolated from *N. alata* are not limited to the digestive zones but were also detected in tissues of the root, stem, leaf, and tendril with the highest levels being recorded in leaf tissues [32]. Athauda et al. [31] argued that these may represent vacuolar APs, different from nepenthesin. This is also evident in our study, wherein the expression of a single transcript (DN48653_c0_g1_i1) was recorded in other zones/parts of the *N. khasiana* leaf, therefore implying that both extracellular and intracellular APs are expressed in the *N. khasiana* leaf.

Additional transcripts (DN31936_c0_g1_i1, DN31936_c0_g1_i2) showing relatively higher expressions in the digestive zone corresponds to the S-like ribonuclease of *N. bicalcarata*. Matthews [33] was the first to report the presence of ribonuclease in the pitcher fluids of several *Nepenthes* species based on experiments that indicated degradation of TMV RNA. Similarly, Stephenson and Hogan [34] reported the presence of ribonuclease activity in pitcher fluid of *N. ventricosa*, and identified a putative RNase clone having 66% identity to S-like RNase. This was further corroborated by a recent study that suggested the probable role of S-like RNases in plant carnivory because of their high expression in the trapping organs of carnivorous plants [35]. In non-carnivorous plants, S-like RNases are induced upon phosphate starvation or mechanical injury [36], probably to promote phosphate mobilization in the event of nutrient limitation. In *N. khasiana*, high expression of the S-like RNase in the digestive zone would have facilitated phosphate mobilization for growth and development. The availability of phosphate in the pitcher fluid is made possible by the action of phosphatases on prey-derived phosphate containing compounds. Phosphatase activity has been detected in the digestive glands of *N. tobaica* and these enzymes are most likely secreted into the pitcher fluid to aid phosphate hydrolysis [37]. Our data showed expression of transcripts sharing homology to acid and protein phosphatases, some of which (DN46307_c0_g2_i1, DN46369_c0_g1_i1, DN46369_c0_g1_i4, DN6769_c0_g2_i1) are highly expressed in the digestive zone, providing additional support to Płachno et al. [37] observations on *N. tobaica*.

The present data also showed the presence of transcripts sharing homology to putative peroxidases. Peroxidases have been identified in a number of *Nepenthes* species and are known to play an important role in plant protection against pathogens [38]. They may also aid in protein degradation via the production of reactive oxygen species [39]. One transcript (DN61358_c1_g2_i4) from our data showed homology to peroxidases isolated from *N. alata* [38]. However, its expression level is low as compared to other putative peroxidases identified in our results (DN42192_c0_g1_i1, DN42192_c0_g1_i2). These putative peroxidases are expressed throughout the *N. khasiana* leaf with higher expression in the digestive zone. This relatively large number of highly expressed putative peroxidases demonstrates the crucial role that peroxidases play in prey digestion and protection against pathogen attack.

Our transcriptome data also points to the presence of transcripts encoding C-terminal peptidases, five of which are expressed at higher levels in the digestive zone (Table 1). C-terminal peptidase represents a class of serine proteases, identified earlier in the digestive fluid of *N. alata* [40]. Interestingly, our analysis of the transcriptome data also indicated the presence of a single transcript (DN3077_c0_g2_i1) showing homology to the cysteine-rich protein defensin. These proteins act mostly against fungi [41].

In a recent study, Rottloff et al. [42] identified 20 new proteins that are possibly secreted into the pitcher fluid of five different *Nepenthes* species. These include serine carboxypeptidases, α- and β-galactosidases, lipid transfer proteins and esterases/lipases. We also show here the presence of transcripts sharing homology to these putative proteins, thereby confirming Rottloff et al. [42] observations. Among them, two transcripts (DN40795_c0_g2_i1, DN40795_c0_g2_i2) encoding serine carboxypeptidase match those identified by Rottloff et al. [42], both of which are expressed exclusively in the digestive zone. Taken together, our findings suggest that *N. khasiana* plants express a broad array of enzymes-encoding genes helpful in the digestion of captured prey, some of which may be representatives of the genus *Nepenthes*.

### Pathogenesis-related (PR) genes are expressed in *N. khasiana* pitchers to inhibit the growth of pathogenic microbes

Once captured prey gets digested, the pitcher fluid becomes a rich source of essential nutrients supporting the growth and development of the plant. In turn, it also favors the growth of bacteria and fungi. Thus, there exists a competition between the *Nepenthes* plant and microbes for the available nutrients in the pitcher fluid [15]. To make full use of the dissolved nutrients, *Nepenthes* plants have adopted a strategy of inhibiting the growth of potential microbial competitors or pathogens through the release of pathogenesis-related (PR) proteins. It was shown earlier that PR proteins such as thaumatin-like proteins and β-1,3-glucanases are secreted into the pitcher fluid of *N. alata* [30]. Our results show the presence of transcripts sharing homology to thaumatin-like proteins and glucanases. Two transcripts (DN60173_c0_g2_i12, DN60173_c0_g2_i2) of the thaumatin-like proteins match those identified earlier in *Nepenthes*, all of which are highly expressed in the digestive zone. Although the expression of a single transcript (DN60686_c0_g1_i1) showing homology to glucanase is expressed at higher levels in the digestive zone, the expressions of two β-1,3-glucanase transcripts (DN24941_c0_g1_i3, DN62573_c0_g1_i5) from *N. khasiana* that matches the one isolated from *N. alata* is relatively low, implying that β-1,3-glucanases are not strongly induced in *N. khasiana.* This may also be the case in *N. alata*, as silver-stained SDS-PAGE gel of Hatano and Hamada [30] points to weak signal thereby corroborating our results. Lipases have also been detected in the pitcher fluids of several *Nepenthes* species [38, 39, 42, 43]; but their role as yet is not clear, although it has been suggested that they promote protein availability through cell membrane disruptions [44]. We detected several transcripts that match GDSL-like lipase, some of which are expressed exclusively in the digestive zone (DN43304_c0_g1_i1, DN43304_c0_g2_i1). GDSL LIPASE-LIKE 1 (GLIP1) has been shown to induce systemic resistance against pathogens in Arabidopsis [45]. Because of its increased expression in the digestive zone, the result points to its role in plant immunity. In addition, we identified a number of transcripts showing homology to genes encoding type III polyketide synthase (PKS) of a carnivorous plant *Drosophyllum lusitanicum*. Type III PKSs are involved in the biosynthesis of secondary metabolites [46], one of which includes naphthoquinones [47]. The exclusive expression of type III PKS genes in the digestive zone of *N. khasiana* suggests that naphthoquinones are required for defense against microbial pathogens. Besides these enzymes, class IV chitinase is another PR protein that aid in plant defense and our results show that it is highly expressed in the pitcher. Based on our results, thaumatin-like proteins, class IV chitinase and GDSL-like lipase provide protection to *N. khasiana* against pathogen attack.

### Transcripts of fungal origin are mostly detected in the digestive zone

The probable role of microbes in prey digestion was brought into limelight following the isolation of bacteria from the fluid of unopened *N. alata* pitchers [48]. This continued to be reported [13, 14], although several other pieces of evidence point to a microbe-free digestive fluid from unopened *Nepenthes* pitchers [12, 15]. We do not attempt to resolve this controversy in the present paper; however, we do feel that a discussion on this aspect is warranted because of the detection of transcripts of microbial origin. Our results show the presence of both bacteria and fungi in the different parts/zones of the *N. khasiana* leaf. This is expected as the tissues were sampled from opened *N. khasiana* pitchers and reports are available that indicated presence of microbe either as endophytes or coming from the phyllosphere [14, 48]. But what’s interesting is the pattern in which these microbes are detected: transcripts of bacterial origin occur mostly in the waxy zone while fungal transcripts are present all along the leaf, the majority of which are detected in the digestive zone. This is in line with the findings of Buch et al. [15], which showed that bacteria die when incubated in the pitcher fluid whereas fungi survived but failed to grow. It also points to the important role fungi plays if at all microbes are involved in prey digestion or competition against available nutrients. Fungal endophytes have been isolated from several *Sarracenia* and *Nepenthes* species [49, 50] and among them, *Aspergillus*, *Colletotrichum*, *Meyerozyma*, *Penicillium* and *Trichoderma* represent some of the genera detected in the present study. *Meyerozyma guilliermondii* is phosphate-solubilizing yeast which promotes nutrient uptake in maize and lettuce and provides protection against fungal pathogens [51]. This evidence points to the existence of a symbiotic relationship between *N. khasiana* and fungi. We do not rule out the role of bacteria in prey digestion as certain bacterial transcripts were detected in the digestive zones. As is the case in the human gut, these might actually help in the digestion of captured prey. In fact, we detected transcripts matching bacteria reported in the human gut, e.g. Bifidobacterium [52]. Surprisingly, only two bacterial species identified in the present study match those that are identified recently from the digestive fluid of a *Nepenthes* species using 16S rDNA and MALDI-TOF MS [53]. These include *Leifsonia aquatica* and *Myroides odoratimimus*. Although Chan et al. [53] did not specify the species name, these results imply that different *Nepenthes* species harbor different microbial communities. This proposition also finds support from the study of Takeuchi et al. [14], which detected only one bacterial operational taxonomic unit (OTU) common among all the 16 *Nepenthes* samples investigated. In 14 samples, however, Takeuchi et al. [14] detected 22 shared OTUs, some of which were also detected in the present study. These include *Acinetobacter*, *Bacillus*, *Micrococcus*, *Pedobacter*, *Pseudomonas*, and *Rhodococcus*.

### Unopened pitchers of *N. khasiana* express several genes involved in prey digestion and protection against pathogen attack

Several reports have pointed to the presence of enzymatic activities in the fluid of unopened *Nepenthes* pitchers. Tökés et al. [44] identified two proteases of different molecular weights and demonstrated the presence of lipase activity in *N. macferlanei*. Several enzymes were also observed in the unopened pitchers of three *Nepenthes* species viz. *N. alata*, *N. tobaica*, and *N. ventricosa* [54]; and in certain species of *Nepenthes*, both unopened and opened pitchers show the same levels of phosphatase activity [55]. Prior to the opening of the pitcher lid, it is likely then that most *Nepenthes* pitchers express several genes involved in prey digestion and protection against pathogen attack. This notion finds support from our analysis of the transcriptome data generated from an unopened *N. khasiana* pitcher. The results indicated that most transcripts sharing homology to key enzymes involved in prey digestion and plant defense are also expressed in the unopened pitcher. For example, nepenthesin I and II are commonly expressed in both opened and unopened pitchers. On the other hand, class IV chitinase is specifically expressed in the opened pitcher (Fig. 6). Our findings suggest that in response to the presence of captured prey or pathogenic microbes, *N. khasiana* plants employ a broader spectrum of digestive enzymes.

### Leaf polarity genes may play a key role in the development of the *Nepenthes* pitcher

Our correlation-based hierarchical clustering analysis of the commonly expressed genes indicated similar expression patterns between the leaf base and the different parts/zones of the pitcher tube. These similarities not only suggest functional relationship but also points to similar cellular processes [56] underlying the development of these two distinct structures i.e. leaf base and the pitcher. Figure 7 shows that the *Nepenthes* pitcher possesses stomata, albeit at reduced number in comparison to the photosynthetically efficient leaf base. This suggests recruitment of the underlying genetic mechanism that governed stomatal development processes. However, their low density as evidenced by SEM micrographs and epidermal imprints indicate reduced expression of genes that positively regulates stomatal development. Assuming that genes known to play a role in the development of stomata in the model plant *Arabidopsis thaliana* are also responsible for such developmental events in *N. khasiana*, we could essentially make predictions of their expression patterns along the *N. khasiana* leaf. Most genes behaved as predicted (Fig. 8), and in cases where they follow the predicted pattern, these are also well-correlated by the qPCR results (Fig. 8a-c). Interestingly, cross sections along the *N. khasiana* leaf depicted leaf base as a typical angiosperm leaf with maintained polarity showing distinct adaxial and abaxial domains (Fig. 7f). However, cells of the pitcher, as well as the lid, are not differentiated into palisade and spongy parenchyma (Fig. 7h-j; also refer Pavlovič et al. [4]), indicating a loss of polarity specification. We recently show that the pitcher of a young *N. khasiana* leaf share anatomical features with the young in-rolled leaf base lamina [28], suggesting that the loss of polarity in the pitcher occurs at later stages of pitcher development. The loss of adaxial-abaxial polarity in the *Nepenthes* pitcher may be associated with reduced *KAN1* expression and/or increased expression of *AS2* as evidenced in Arabidopsis, whereby leaf anatomy of loss-of-function *kan1* mutants and *AS2* overexpressed plants displayed disrupted adaxial-abaxial polarity as compared to wild-type [57, 58]. We extended our investigation into genes involved in leaf polarity specification and determined their expression pattern along the *N. khasiana* leaf. However, the RNA-seq derived expression pattern of *NkKAN1* and *NkAS2* genes do not follow the predicted pattern (Fig. 8g, l); rather, expression of the class III *HOMEODOMAIN-LEUCINE ZIPPER* (HD-ZIPIII) and *ARGONAUTE* (*AGO*) genes were upregulated in the tendril (Fig. 8e, f, o, p). Previous reports have shown that the constitutive expression of the adaxial *HD-ZIPIII* genes *PHB* and *REV* transformed the flat ovate-shaped leaf of *Arabidopsis* into rod- or trumpet-shaped leaves [59–61]. These leaves show an abundance of trichomes on the epidermal surfaces, characteristics of an adaxialized leaf phenotype [62]. Besides showing increased *PHB/REV* expression, the tendril of *N. khasiana* is made up of relatively higher number of trichomes (Figs. 1 and 7b), in line with those observed in the leaves of the Arabidopsis *phb1-d* mutant [62]. Trichomes were also observed on the abaxial surfaces of the *N. khasiana* pitcher comprising the digestive zone, waxy zone and lid (Fig. 7c-e).

## Conclusions

In the present study, we analyzed and report the transcriptome data of the highly specialized *N. khasiana* leaf comprising the leaf base lamina, tendril and the different parts/zones of the pitcher tube viz. digestive zone, waxy zone and lid. We found that irrespective of whether the pitcher lid is opened or closed, many of the enzyme-encoding genes involved in prey digestion and plant defense are commonly expressed. This observation suggests that *Nepenthes* plants equip themselves with the necessary machinery required for prey attraction, capture and digestion prior to the opening of the pitcher lid. In addition, our findings imply that fungi may play a crucial role over bacteria in the digestion of captured prey if at all microbes are involved, and may exist as symbionts of the *Nepenthes* plants. Furthermore, we show that the adaxial/abaxial polarity is maintained in the leaf base but become disrupted in the different parts/zones comprising the pitcher tube. Our examination of the RNA-seq derived expression patterns of a number of genes known to play a role in leaf polarity specification suggest that class III *HD-ZIP* genes may play a key role in the development of the *Nepenthes* pitcher.

## Methods

### Plant material and tissue harvesting

*Nepenthes khasiana* plants used in the present study are found in the wild located at Jaraiñ, Jaiñtia Hills District, Meghalaya (25° 18.651˝ N, 92° 07. 786˝ E). Tissues representing the five different parts/zones of the *N. khasiana* leaf viz. leaf base, tendril, digestive zone, waxy zone and lid were harvested at two separate dates, one in the month of June 2013 and another in October 2013 (Additional file 1: Fig. S14). We felt that collecting samples at two different dates will not affect the results as these samples represent vegetative tissues of the same kind; rather, this sampling strategy would add more strength to our findings. Mature leaves were selected having developed pitchers characterized by an opened lid and the presence of captured, killed insects in the digestive fluid. The fluid was drained off prior to tissue freezing. Tissue samples were then frozen in liquid nitrogen, transferred into dry ice, transported to Jawaharlal Nehru University, New Delhi and kept at − 80 °C, until further processing. Tissue samples were collected by Jeremy Dkhar on two separate trips. Identification of the plant species was made by Jeremy Dkhar based on reliable sources available in the literature. A voucher specimen having a deposition number 17466 has been deposited in the Herbarium section of CSIR-Institute of Himalayan Bioresource Technology, Palampur, India.

### Histological and scanning electron microscopy (SEM) analyses

Small tissue pieces of each part/zone of the *N. khasiana* leaf were fixed in 0.1 M phosphate buffer containing 2.5% glutaraldehyde (pH 7.2). Fixed tissue samples were dehydrated through a graded ethanol series, embedded in saturated paraffin wax, cross-sectioned, de-waxed in xylene, stained with 0.05% toluidine blue and viewed and photographed using a Nikon Eclipse Ti-S Inverted Microscope available at Central Instrumentation Facility, School of Life Sciences, JNU, New Delhi. For SEM, samples were processed at Sophisticated Analytical Instrumentation Facility, North-Eastern Hill University, Shillong. Fixed samples were washed in buffer overnight, post fixed in 1% osmium tetraoxide, dehydrated through increasing concentrations of acetone, dried using an HCP-2 (Hitachi) critical point-drier, coated with gold and viewed under JEOL JSM-6360 SEM.

### RNA extraction, library preparation and sequencing

We extracted total RNA from all five distinct parts/zones of the *N. khasiana* leaf using Raflex Kit (Bangalore Genei, India) and/or Spectrum Plant Total RNA Kit (Sigma, USA) as per the instructions of the manufacturers. Quality check of the extracted RNA samples was performed using the Agilent 2100 Bioanalyzer. Extracted RNAs showed three distinct bands (28S, 18S and 5S) on EtBr-stained formaldehyde agarose gel (Additional file 1: Fig. S15). RNA integrity number (RIN) values of the extracted RNAs ranged from 6.6–8.5. Library preparation and sequencing were performed at the Centre for Cellular and Molecular Platforms, Bangalore, India. One microgram of RNA each was used for library preparation and mRNA was purified using the polydT Oligo beads. The mRNA was then fragmented followed by cDNA synthesis. End repair, A-Tailing and adapter ligation were performed followed by PCR enrichment for 15 cycles. The Agilent Bioanalyzer was used to validate the generated libraries and sequencing was performed on an Illumina HiSeq 1000 platform as per the recommended protocol of the manufacturer, to generate 2 × 100 bp paired-end data. The five different parts/zones of the *N. khasiana* leaf were harvested from two separate individual plants and sequenced separately. Thus, two biological replicates were used for RNA-seq.

### Data pre-processing and de-contamination

The data pre-processing and de-contamination steps were performed as mentioned in Dkhar and Pareek [28].

### De novo transcriptome assembly, annotation and differential expression analysis

Transcriptome reads were pooled into a single data set and de novo assembled using Trinity [18, 19], applying the default settings. We then aligned individual reads from each tissue i.e. leaf base, tendril, digestive zone, waxy zone and lid to the assembled reference transcriptome to estimate gene expression using Bowtie2 version 2.2.2. We allowed up to 1-mismatches in the seed region (length = 31 bp) and all multiple mapped position were reported. The FPKM values were calculated using SciGenom Labs Pvt. Ltd. Perl script. The assembled transcripts were then annotated using a SciGenom Labs Pvt. Ltd. pipeline CANoPI (Contig Annotator Pipeline). Transcripts with FPKM ≥1 and minimum length ≥ 200 were selected for annotation against the NCBI non-redundant protein database using BLASTX 2.2.28 program [20] with E-value cut-off ≤10^− 5^ and similarity score ≥ 40%. BLASTX hits transcripts were also annotated against the UniProt database. Transcripts with read count ≥1 were selected for differential gene expression analysis using DESeq 3.2.0 [22]. The analysis was carried out in pairwise combinations between parts/zones - for example leaf base vs. tendril, tendril vs. leaf base, leaf base vs. digestive zone, and so on - to identify upregulated and downregulated genes in each part/zone. Transcripts with adjusted *p*-value (FDR) ≤ 0.05 and log fold change (logFC) ≥ 1 or ≤ − 1 were considered significantly differentially expressed.

### Correlation analysis

We used the freely available R software (https://www.r-project.org/) to perform the correlation analysis among the five different parts/zones of the *N. khasiana* leaf as mentioned in Dkhar and Pareek [28].

### Overrepresentation analysis

Prior to running overrepresentation analysis, a mapping file of the DEGs was generated using the automated annotation software Mercator [23]. Here, all DEGs sequences were used as input sequences and uploaded as a single file in fasta format into the Mercator web application available at http://www.plabipd.de/portal/web/guest/mercator-sequence-annotation. Each input sequence is expected to map to one or more MapMan bins. A Fisher’s exact test followed by the Benjamini-Hochberg correction as implemented in PageMan software [24] was used to identify functional MapMan categories with significant differences among the different parts/zones of the *N. khasiana* leaf.

### K-means clustering and functional category enrichment analyses

Prior to clustering, the number of clusters k was estimated using the Figures of Merit (FOM) application embedded in the MeV program [26]. The FPKM values were added throughout by 1 and log_2_ transformed before running FOM. Five runs were performed and all runs showed similar results. In addition to FOM, the gap statistic algorithm [63] in R (https://www.r-project.org/) was also employed to estimate the number of clusters k. After estimating k (k = 4), we then performed k-means clustering of the significantly DEGs (log_2_ transformed FPKM) using the K-means / K-medians Support (KMS) module in MeV and applying the Kendall tau rank correlation. KMS was performed four times. Functional MapMan category enrichment analysis for each cluster was performed by applying the Wilcoxon statistics and Benjamini Hochberg multiple testing correction as implemented in Pageman [24].

### Identifying genes involved in prey digestion and plant defense

We manually searched the list of annotated transcripts showing significant differential expressions to identify genes involved in prey digestion and protection against pathogen attack. We then checked their abundance and compared their expression patterns among the five different parts/zones of the *N. khasiana* leaf.

### Transcript profiling of key regulatory genes involved in plant development

Similarly, we examined the expression levels of key regulatory genes involved in stomatal development and leaf polarity specification. We extended our investigation into the total list of annotated transcripts to identify genes of interest and compared their expression patterns among the five different parts/zones of the *N. khasiana* leaf.

### Validation of RNA-seq data using real-time qPCR

The RNA-seq results were validated using real-time qPCR. cDNAs of four biological replicates were synthesized using the First Strand cDNA synthesis kit (Thermo Scientific). Transcripts for validation were selected from a list of identified genes known to play a role in prey digestion and plant defense, stomatal development and leaf polarity. In addition, eight randomly selected genes were also used for validation. We initially tested four endogenous genes {Actin, Elongation factor (*ELF*), Glyceraldehyde-3-phosphate dehydrogenase (*GAPC2*) and Ubiquitin (*UBQ*)} for stable expression, out of which one (*ELF*) was used for the normalization of the expression of the selected genes. Real-time qPCR assays were carried out as mentioned in Dkhar and Pareek [28]. Three technical replicates were analyzed for each sample and data analysis was performed using 7500 Software v 2.0.5 (Applied Biosystems). The 2^-ΔCT^ values were generated and these were then compared with the RNA-seq derived FKPM values. ANOVA of SPSS was performed to test the significance level (*p* < 0.05) and Pearson correlation (r) was calculated to check for correlation between the RNA-seq and qPCR data. Table S3 in Additional file 1 contains the list of qPCR primers.

### Identifying genes involved in prey digestion and plant defense in the unopened pitchers of *N. khasiana*

Unopened pitchers of *Nepenthes* are known to contain digestive fluid, though the amount is lesser when compared to the fluid of opened *Nepenthes* pitchers [64]. It may also contain protein at very low levels, which increases upon opening of the pitcher lid, or at levels identical to those of an opened pitcher [55]. In such a scenario, it is expected then that the unopened pitchers of *Nepenthes* should show the expression of genes involved in prey digestion and plant defense. To confirm, we examined the list of significantly DEGs of an unopened *N. khasiana* pitcher to identify genes involved in prey digestion and protection against pathogen attack. We then compared them with those genes identified from the opened pitchers of *N. khasiana*. The transcriptome data of the unopened *N. khasiana* pitcher was generated independently and with a different objective from the present study [28] and can be accessed online at NCBI under accession numbers SRR4340048 (raw reads) and GFDV00000000 (assembled transcript sequences). All bioinformatic analysis steps leading to the identification of DEGs in the unopened *N. khasiana* pitcher were performed as mentioned above.

## Supplementary information

**Additional file 1: Fig. S1.** Length distribution of the assembled transcripts of *N. khasiana* leaf. **Fig. S2.** **a**, E-value distribution of BLASTX hits of *N. khasiana* leaf transcriptome against the NCBI non-redundant protein database. **b**, BLASTX similarity score distribution of *N. khasiana* leaf transcriptome with the NCBI non-redundant protein database. **Fig. S3.** Top 50 organisms distribution of the assembled transcriptome using BLASTX. **Fig. S4.** Metabolic pathway mapping of the assembled transcriptome. **Fig. S5.** Top 10 GO terms in biological processes, cellular components and molecular functions. **Fig. S6.** Over-represented and under-represented molecular functions in each of the five different parts/zones of the *N. khasiana* leaf. No enrichment was detected in the lid. **Fig. S7.** Transcript expression distribution in the five tissue samples. **Fig. S8.** Assigning Mapman ‘bins’ to the DEGs using the automated annotation software Mercator available online at https://mapman.gabipd.org/app/mercator. **Fig. S9.** Determining the number of clusters for k-means clustering using the Figures of Merit (FOM) application embedded in the MeV program. The adjusted FOM decreases sharply and levels out after reaching 4 clusters. **Fig. S10.** Determination of the number of clusters for k-means clustering using the gap statistic algorithm in R. The number of clusters is 6. **Fig. S11.** *N. khasiana* plant showing several developing leaves, each attaining distinct stages of development. Stage 5 represents the leaf (L5) showing pitcher expansion with the lid remaining unopened. Transcriptome data of stage 5 was included in the present study. White vertical/horizontal lines specify the dissected regions of each stage. Bar = 6 cm. **Fig. S12.** Relative abundance of bacterial transcripts against fungal transcripts across the different parts/zones of the *N. khasiana* leaf. LB: leaf base; T: tendril; D: digestive zone; W: waxy zone; L: lid. **Fig. S13.** Epidermal nail polish imprints of the abaxial surfaces of four different parts/zones of the *N. khasiana* leaf. These imprints are then used to estimate stomatal density [1]. Arrow head denotes stomata. **Fig. S14.** Sample collection for transcriptome sequencing of *N. khasiana* leaf. **a**, *Nepenthes khasiana* plants growing in the natural habitat at Jaraiñ, Jaiñtia Hills District, Meghalaya. **b**, mature pitcher with fully opened-lid and prominent wings formed along the sides of the pitcher. **c-d**, preparation of the different parts/zones of the leaf viz. leaf base, tendril, digestive zone, waxy zone and lid for preservation in liquid nitrogen. Note: transition regions represent those regions in the leaf that indicate a shift from one part/zone to another. **Fig. S15.** Extraction and quality check of total RNA from *N. khasiana* leaf. **a**, total RNA extracted from five different parts/zones of the *N. khasiana* leaf. The full-length gels photos are provided in Fig. S16. below; **b**, total RNA profile and the corresponding peaks resulting from a quality check of the isolated total RNA (leaf base) using an Agilent Bioanalyzer; **c**, electropherogram profile of RNA library. **Fig. S16.** Full-length gels photos of the total RNAs isolated from the five different parts/zones of the *N. khasiana* leaf. The gel photo on the left shows the extracted RNAs of the digestive zone, tendril and the leaf base (lanes 4, 5 and 6). Since the RNA of the tendril is of poor quality (lane 5), isolation was repeated to yield a better one (lane 8 of middle gel photo). The gel photo on the right shows the extracted RNAs of the lid and the waxy zone (lanes 4 and 5). The RNA gel images of each part/zone were cropped and presented in Fig. S15a. **Note S1.** Stomatal density and distribution in the model plant *Arabidopsis thaliana*. **Table S1.** RNA sequencing read statistics of the different tissue parts of two *N. khasiana* leaf samples. We performed MD5 CheckSum on the FASTQ files under each category (raw and quality filter passed reads) to ensure data integrity. **Table S2.** Alignment summary of the individual reads to the reference transcriptome. **Table S3**. List of qPCR primers used for the validation of RNA-seq derived transcript expression pattern.

**Additional file 2: Table S4.** List of transcripts encoding enzymes involved in prey digestion and plant defense in *N. khasiana*.

**Additional file 3: Table S5.** List of transcripts encoding enzymes involved in prey digestion and plant defense detected in the unopened pitchers of *N. khasiana*.

**Additional file 4: Table S6.** List of transcripts of bacterial origin.

**Additional file 5: Table S7.** List of transcripts of fungal origin.

**Additional file 6: Table S8.** List of transcripts of bacterial origin detected in the unopened pitchers of *N. Khasiana*.

**Additional file 7: Table S9.** List of transcripts of fungal origin detected in the unopened pitchers of *N. khasiana*.

## Data Availability

The datasets generated and analyzed during the current study are available in the NCBI Short Read Archive under accession number SRP064181.

## Abbreviations

AGO: ARGONAUTE
DEGs: Differentially expressed genes;
FPKM: Fragments per kilobase of transcript per million mapped reads
*HD-ZIPIII*: Class III *HOMEODOMAIN-LEUCINE ZIPPER*
*REV*: *REVOLUTA*
SEM: Scanning electron microscopy
