# Supplementary material for: Pitchers of Nepenthes khasiana express several digestive-enzyme encoding genes, harbor mostly fungi and probably evolved through changes in the expression of leaf polarity genes
Source: BMC Plant Biol. 2020 Nov 17;20:524. doi: 10.1186/s12870-020-02663-2 (PMC7672872; doi:10.1186/s12870-020-02663-2)
Supplement: Supplementary file 1 — Additional file 1: Fig. S1. Length distribution of the assembled transcripts of N. khasiana leaf. Fig. S2. a, E-value distribution of BLASTX hits of N. khasiana leaf transcriptome against the NCBI non-redundant protein database. b, BLASTX similarity score distribution of N. khasiana leaf transcriptome with the NCBI non-redundant protein database. Fig. S3. Top 50 organisms distribution of the assembled transcriptome using BLASTX. Fig. S4. Metabolic pathway mapping of the assembled transcriptome. Fig. S5. Top 10 GO terms in biological processes, cellular components and molecular functions. Fig. S6. Over-represented and under-represented molecular functions in each of the five different parts/zones of the N. khasiana leaf. No enrichment was detected in the lid. Fig. S7. Transcript expression distribution in the five tissue samples. Fig. S8. Assigning Mapman ‘bins’ to the DEGs using the automated annotation software Mercator available online at https://mapman.gabipd.org/app/mercator. Fig. S9. Determining the number of clusters for k-means clustering using the Figures of Merit (FOM) application embedded in the MeV program. The adjusted FOM decreases sharply and levels out after reaching 4 clusters. Fig. S10. Determination of the number of clusters for k-means clustering using the gap statistic algorithm in R. The number of clusters is 6. Fig. S11. N. khasiana plant showing several developing leaves, each attaining distinct stages of development. Stage 5 represents the leaf (L5) showing pitcher expansion with the lid remaining unopened. Transcriptome data of stage 5 was included in the present study. White vertical/horizontal lines specify the dissected regions of each stage. Bar = 6 cm. Fig. S12. Relative abundance of bacterial transcripts against fungal transcripts across the different parts/zones of the N. khasiana leaf. LB: leaf base; T: tendril; D: digestive zone; W: waxy zone; L: lid. Fig. S13. Epidermal nail polish imprints of the abaxial surfaces of four different [file 12870_2020_2663_MOESM1_ESM.docx]

Additional figures, note and tables for

**Pitchers of *Nepenthes khasiana* express several digestive-enzyme encoding genes, harbor mostly fungi and probably evolved through changes in the expression of leaf polarity genes**

Jeremy Dkhar^1,2^, Yogendra Kumar Bhaskar^3^, Andrew Lynn^3^, Ashwani Pareek^1^

^1^Stress Physiology & Molecular Biology Laboratory, School of Life Sciences, Jawaharlal Nehru University, New Delhi 110067

^2^Agrotechnology Division, CSIR-Institute of Himalayan Bioresource Technology, Palampur 176061, Himachal Pradesh

^3^School of Computational and Integrative Sciences, Jawaharlal Nehru University, New Delhi 110067

Authors email addresses:

jeremydkhar@gmail.com,

2014in-yogendra@qibebt.ac.cn,

andrew@jnu.ac.in

ashwanip@mail.jnu.ac.in

Corresponding author: Jeremy Dkhar


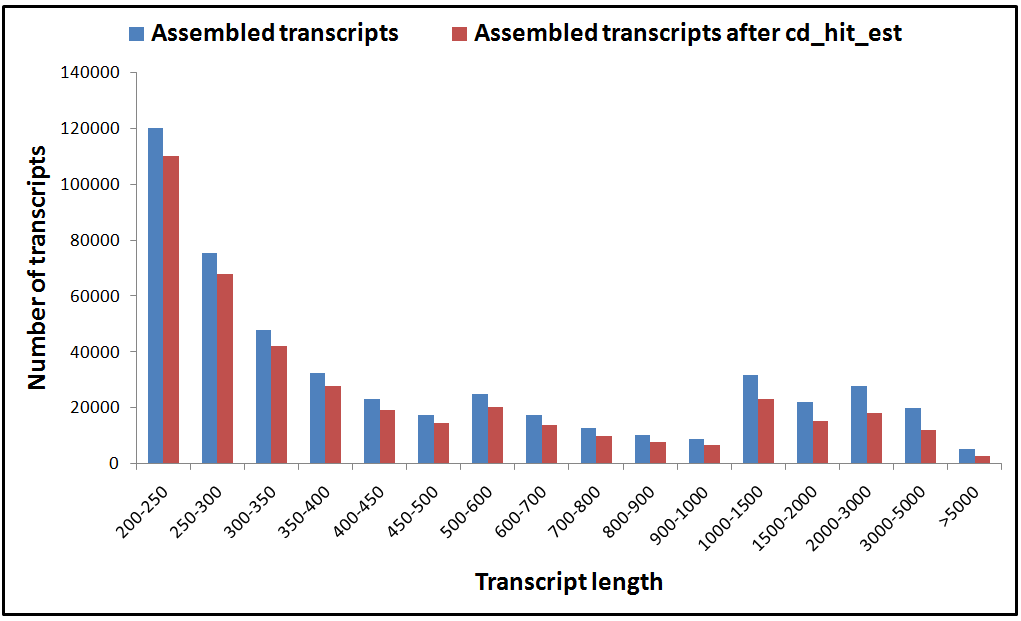


**Fig. S1**. Length distribution of the assembled transcripts of *N. khasiana* leaf.


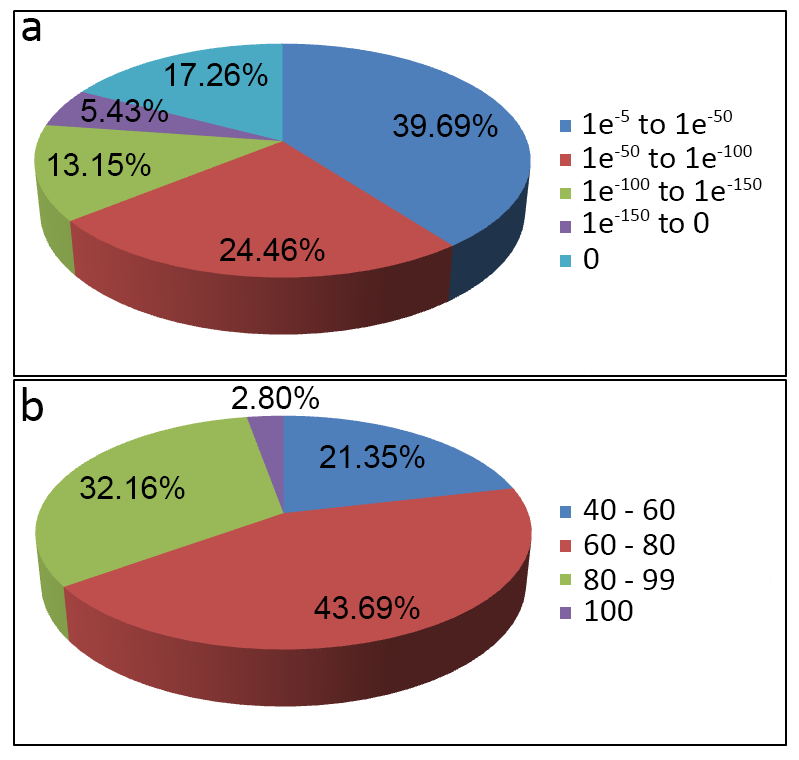


**Fig. S2**. **a**, E-value distribution of BLASTX hits of *N. khasiana* leaf transcriptome against the NCBI non-redundant protein database. **b**, BLASTX similarity score distribution of *N. khasiana* leaf transcriptome with the NCBI non-redundant protein database.


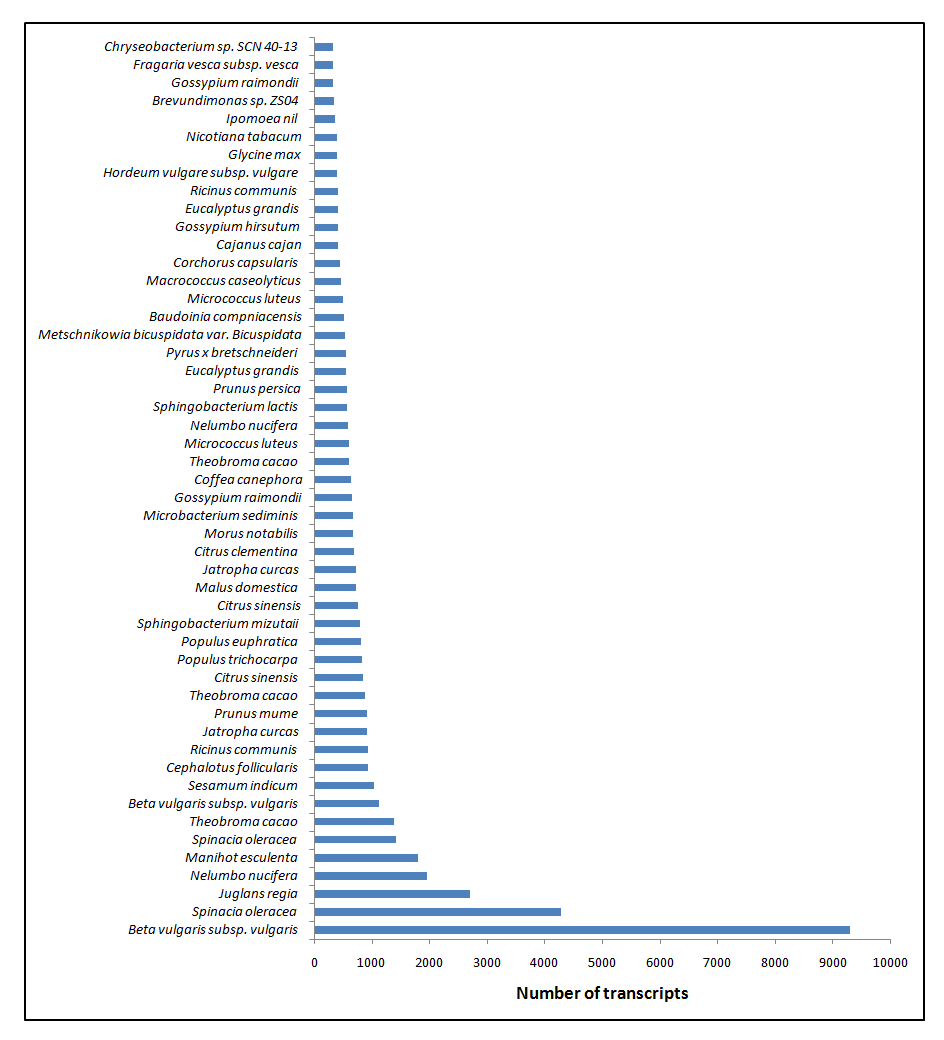


**Fig. S3**. Top 50 organisms distribution of the assembled transcriptome using BLASTX.


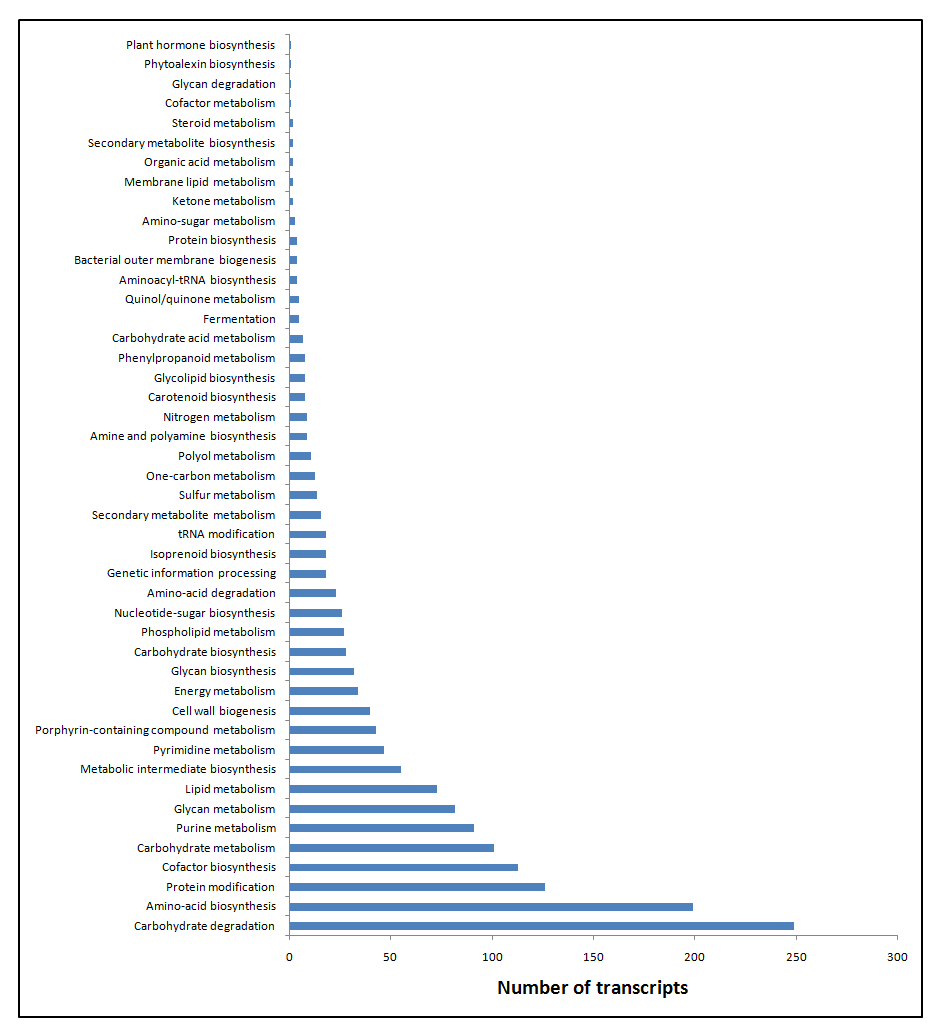


**Fig. S4**. Metabolic pathway mapping of the assembled transcriptome.


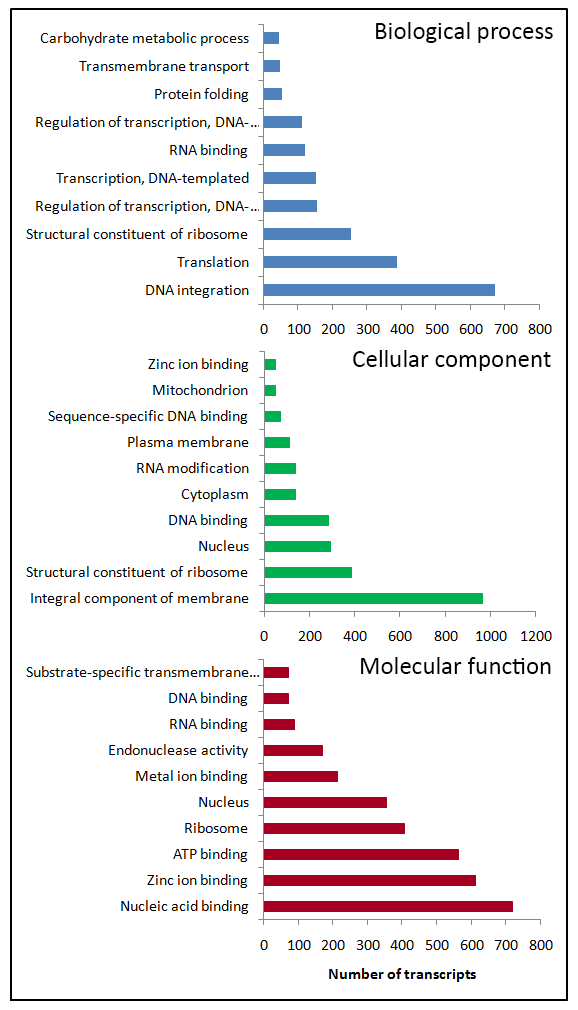


**Fig. S5**. Top 10 GO terms in biological processes, cellular components and molecular functions.


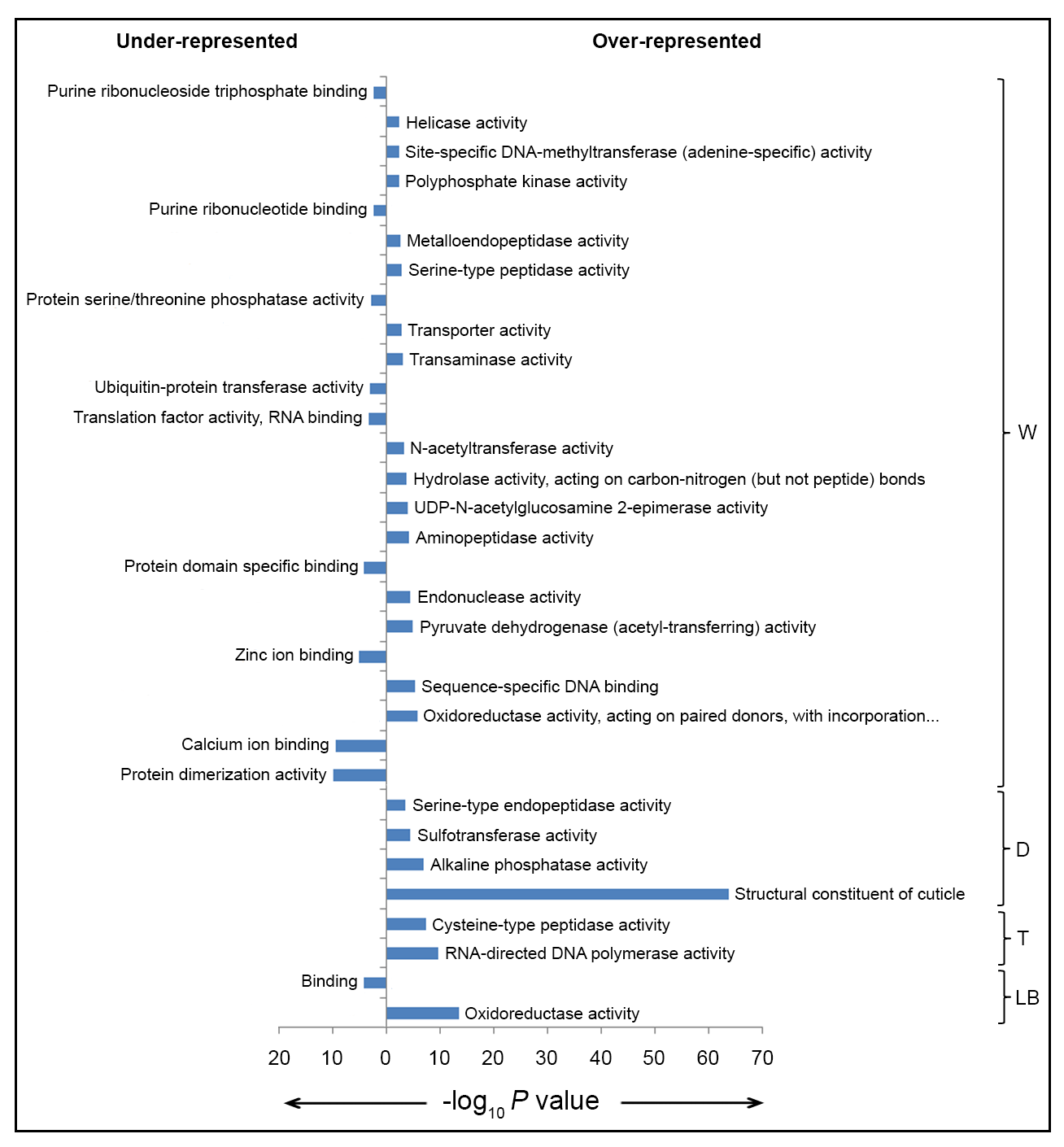


**Fig. S6**. Over-represented and under-represented molecular functions in each of the five different parts/zones of the *N. khasiana* leaf. No enrichment was detected in the lid.


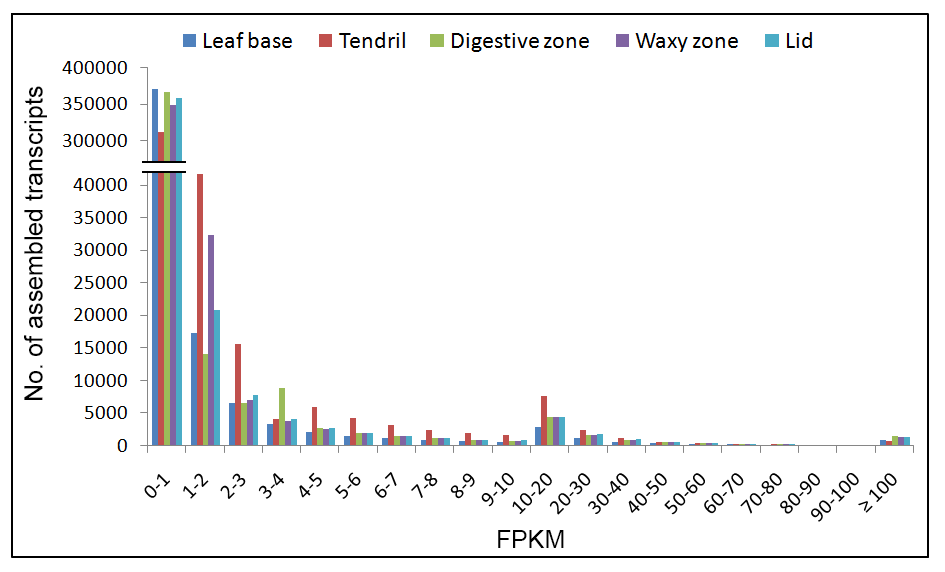


**Fig. S7**. Transcript expression distribution in the five tissue samples.


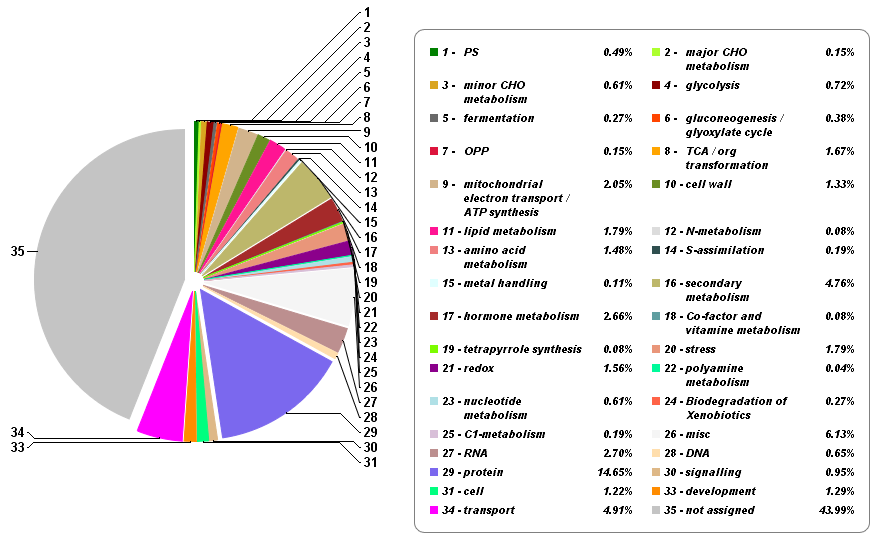


**Fig. S8**. Assigning Mapman 'bins' to the DEGs using the automated annotation software Mercator available online at https://mapman.gabipd.org/app/mercator.


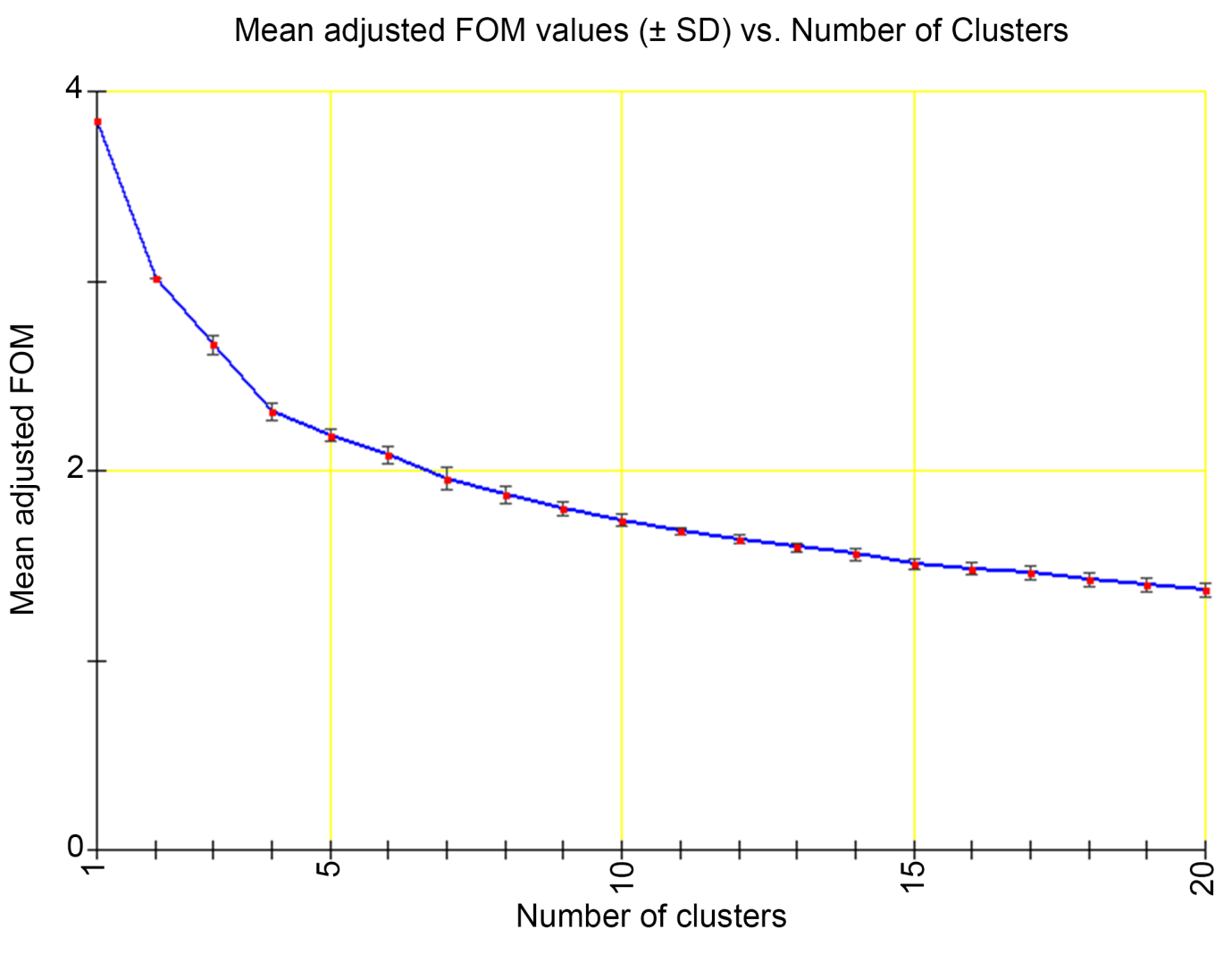


**Fig. S9**. Determining the number of clusters for k-means clustering using the Figures of Merit (FOM) application embedded in the MeV program. The adjusted FOM decreases sharply and levels out after reaching 4 clusters.


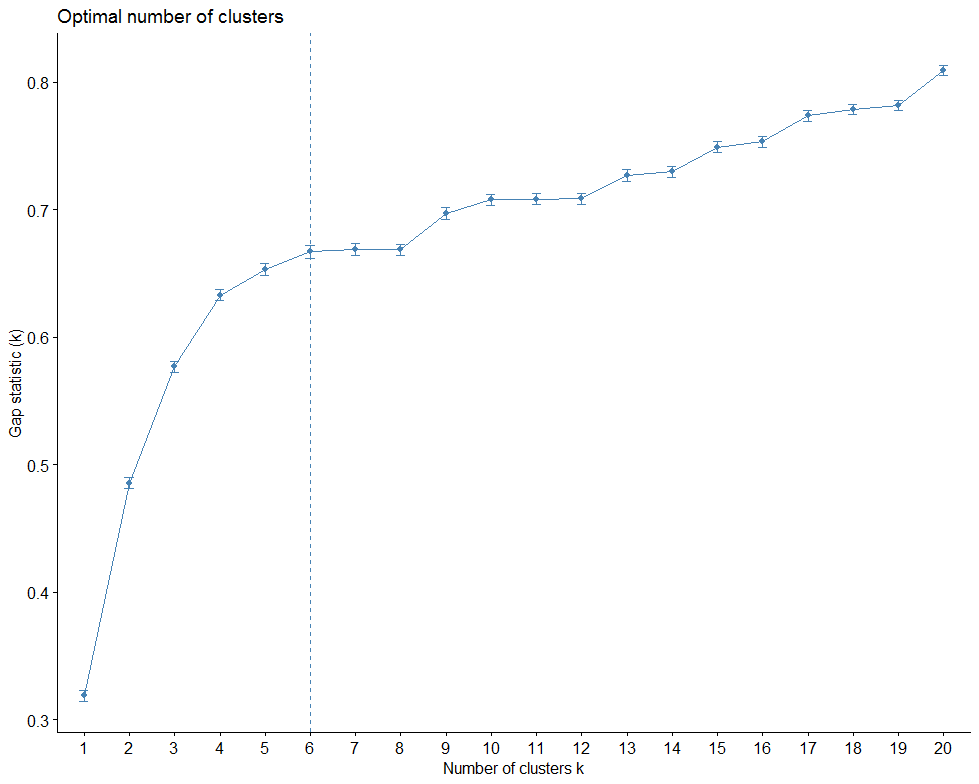


**Fig. S10**. Determination of the number of clusters for k-means clustering using the gap statistic algorithm in R. The number of clusters is 6.


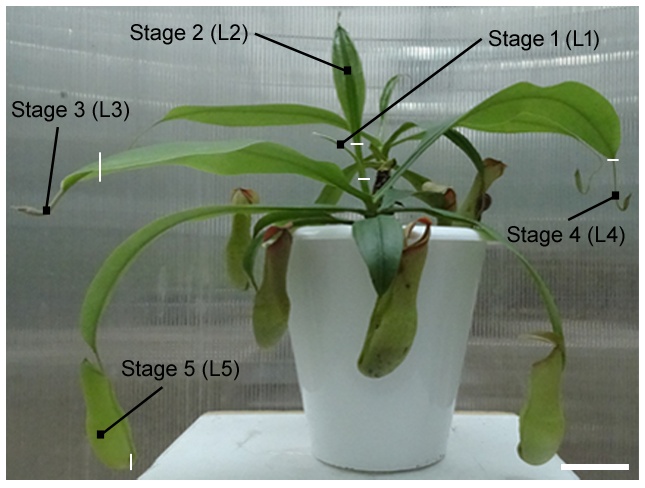


**Fig. S11**. *N. khasiana* plant showing several developing leaves, each attaining distinct stages of development. Stage 5 represents the leaf (L5) showing pitcher expansion with the lid remaining unopened. Transcriptome data of stage 5 was included in the present study. White vertical/horizontal lines specify the dissected regions of each stage. bar=6cm.

**Fig. S12**. Relative abundance of bacterial transcripts against fungal transcripts across the different parts/zones of the *N. khasiana* leaf. LB: leaf base; T: tendril; D: digestive zone; W: waxy zone; L: lid.

**
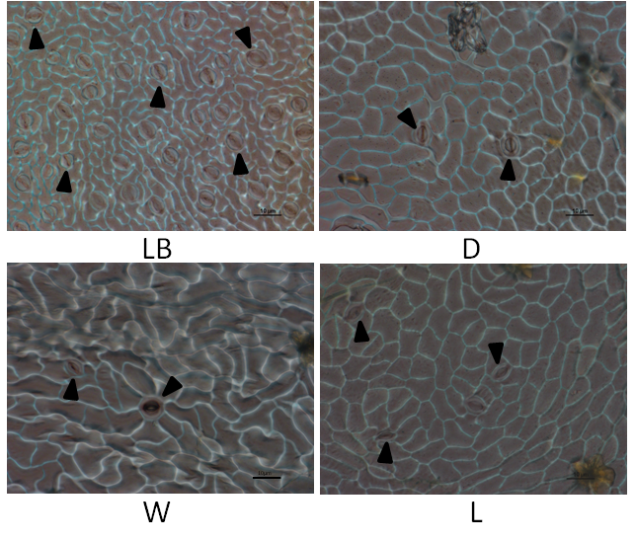
**

**Fig. S13**. Epidermal nail polish imprints of the abaxial surfaces of four different parts/zones of the *N. khasiana* leaf. These imprints are then used to estimate stomatal density [1]. Arrow head denotes stomata.


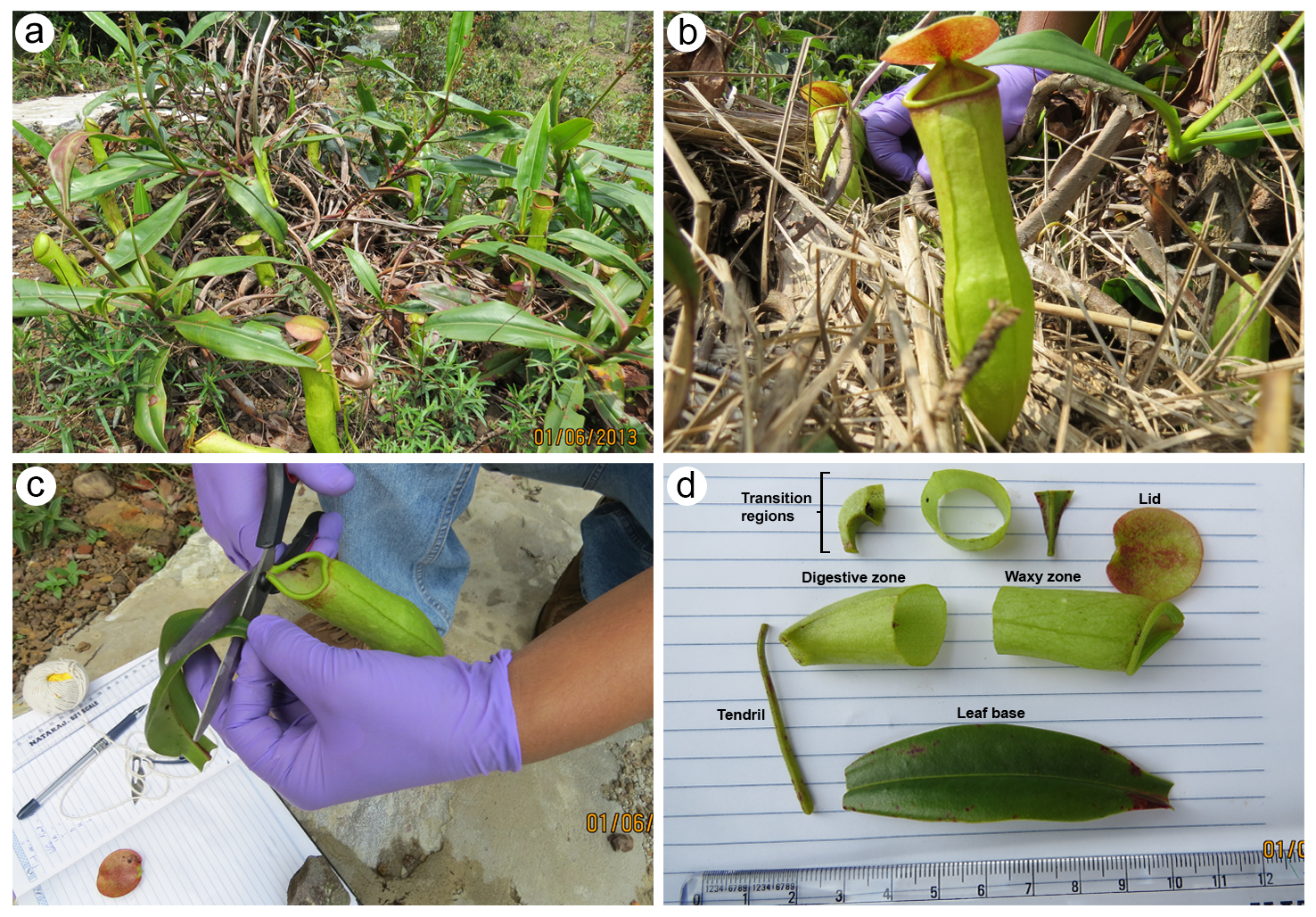


**Fig. S14**. Sample collection for transcriptome sequencing of *N. khasiana* leaf. **a**, *Nepenthes khasiana* plants growing in the natural habitat at Jaraiñ, Jaiñtia Hills District, Meghalaya. **b**, mature pitcher with fully opened-lid and prominent wings formed along the sides of the pitcher. **c-d**, preparation of the different parts/zones of the leaf viz. leaf base, tendril, digestive zone, waxy zone and lid for preservation in liquid nitrogen. Note: transition regions represent those regions in the leaf that indicate a shift from one part/zone to another.


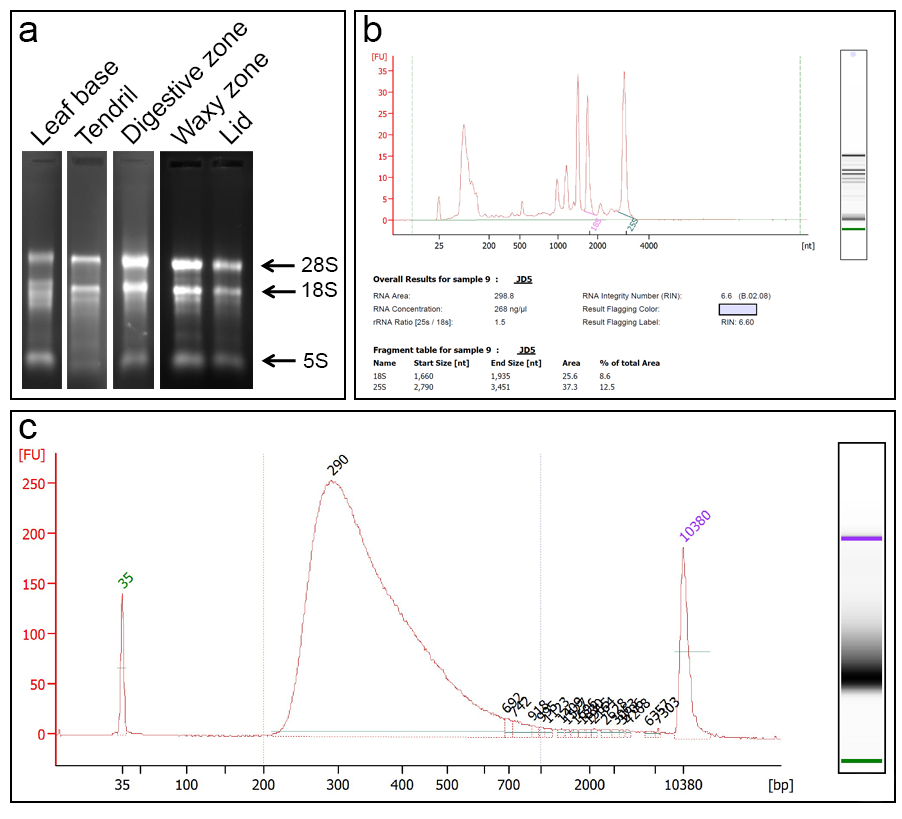


**Fig. S15**. Extraction and quality check of total RNA from *N. khasiana* leaf. **a**, total RNA extracted from five different parts/zones of the *N. khasiana* leaf. The full-length gels photos are provided in Fig. S16 below; **b**, total RNA profile and the corresponding peaks resulting from a quality check of the isolated total RNA (leaf base) using an Agilent Bioanalyzer; **c**, electropherogram profile of RNA library.

**

**

**Fig. S16**. Full-length gels photos of the total RNAs isolated from the five different parts/zones of the *N. khasiana* leaf. The gel photo on the left shows the extracted RNAs of the digestive zone, tendril and the leaf base (lanes 4, 5 and 6). Since the RNA of the tendril is of poor quality (lane 5), isolation was repeated to yield a better one (lane 8 of middle gel photo). The gel photo on the right shows the extracted RNAs of the lid and the waxy zone (lanes 4 and 5). The RNA gel images of each part/zone were cropped and presented in Fig. S15a.

**Note S1**

*Stomatal density and distribution in the model plant Arabidopsis thaliana*

Stomata are specialized epidermal structures that facilitate gas and water-vapour exchanges between plants and the environment. Their number and distribution on the leaf surfaces are regulated during leaf development by both genetic and environmental factors [2]. In the *Arabidopsis* leaf, stomata are arranged in a non-random manner and are constantly separated from one another by at least one intervening cell [3]. Studies have shown that stomatal development and patterning in *Arabidopsis* are controlled by cell-cell signalling genes *TOO MANY MOUTHS* (*TMM*) [2] and *STOMATAL DENSITY AND DISTRIBUTION* (*SDD1*) [4], leucine rich repeat (LRR) receptor-like kinases *ERECTA (ER), ERECTA-LIKE 1 (ERL1) and ERL2* [5] and mitogen-activated protein (MAP) kinase family which includes *MPK3/MPK6*, *MKK4/MKK5* [6] and *YODA (YDA)*, an MAPKK kinase [7]. Functioning upstream of this signalling cascade, and independent of *SDD1*, are the secretory peptides *EPIDERMAL PATTERNING FACTOR* 1 (*EPF1*) AND *EPF2* [8, 9]. Another *EPF* member *STOMAGEN* (*EPF*-like 9) acts as a positive regulator of stomatal development [10]. In addition, three bHLH transcription factors *SPEECHLESS* (*SPCH*)*, MUTE* and *FAMA* and two paralogous R2R3-MYB transcription factors *FOUR LIPS (FLP)* and *MYB88* control cell-fate transition and late-stage mitotic division during stomatal development, respectively [11, 12, 13, 14]. These bHLH proteins require direct interaction with two bHLH-leucine zipper (bHLH-LZ) proteins *ICE1/SCREAM1* and *SCREAM2* to specify their sequential actions [15]. These studies further indicate that the loss-of-function mutation in *SDD1*, *YDA*, *EPF1* and *EPF2* increase stomatal density while mutation in genes encoding *TMM*, *ER*, *ERLs*, *FLP* and MAP kinases cause stomatal clustering [7, 8, 9, 11, 16]. The above-ground epidermis of plants with loss-of-function *SPCH, MUTE* and *FAMA* mutations are devoid of stomata; however, excess stomata are produced on the epidermal surfaces when *SPCH*, *MUTE* and *FAMA* are overexpressed [13]. Overexpression of *YDA* and *MPK3/MPK6* leads to the complete loss of stomata while reduced stomatal density occurs when *SSD1*, *EPF1* and *EPF2* are overexpressed [7, 8, 9, 11, 16]. The loss- and gain-of-function *STOMAGEN* phenotypes showed decreased and increased stomatal densities, respectively [10]. The current model suggests that a bi-directional signalling system involving *STOMAGEN* as a positive signal and *EPF1/2* as negative signal are perceived by receptor components (*ER*, *ERL1/2*, *TMM*) and transmitted to the transcriptional regulators via a MAPK cascade to regulate stomatal density and patterning (Fig. N1).


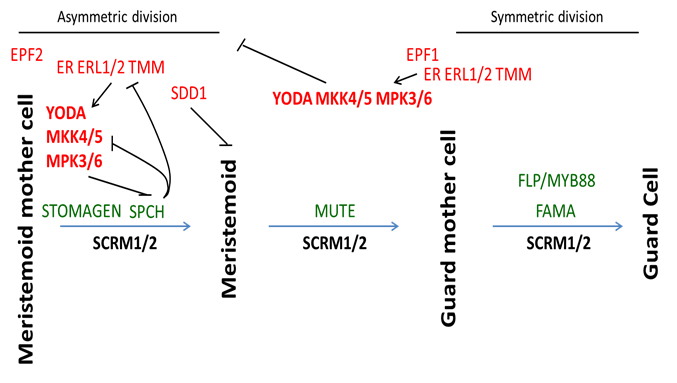


**Fig. N1**. Mechanism of stomatal development in the model plant *Arabidopsis thaliana* (genes in bold were selected for validation using qPCR).

**Table S1**. RNA sequencing read statistics of the different tissue parts of two *N. khasiana* leaf samples. We performed MD5 CheckSum on the FASTQ files under each category (raw and quality filter passed reads) to ensure data integrity.

| Tissue | Total number of paired-end raw reads | Total number of paired-end reads passing quality filter | Yield  (Mbases) | % of ≥ Q30  Bases | MD5 value on raw reads | MD5 value on quality filter passed reads |
| --- | --- | --- | --- | --- | --- | --- |
| *Sample 1* | | | | | | |
| Leaf base | 40,683,261 | 39,148,056 | 7,908 | 90.4 | db3a2cda6a9070937bc13650c551526d | 4ebf12cb4e2233b1d26e70ad9645d81f |
| Tendril | 36,751,633 | 35,261,164 | 7,123 | 93.24 |  |  |
| Digestive zone | 49,164,507 | 47,190,247 | 9,532 | 92.46 |  |  |
| Waxy zone | 31,141,329 | 30,171,870 | 6,095 | 92.45 |  |  |
| Lid | 29,230,170 | 28,288,959 | 5,714 | 92.47 |  |  |
| *Sample 2* | | | | | | |
| Leaf base | 13,460,208 | 13,227,120 | 2,672 | 96.06 | 0ddf974e90bf0cb250fe451f7e6c5dc1 | da523f0975d6dd1061df00f853d6eeb1 |
| Tendril | 20,653,789 | 20,367,239 | 4,114 | 97.09 |  |  |
| Digestive zone | 15,053,763 | 14,838,985 | 2,997 | 96.18 |  |  |
| Waxy zone | 19,281,552 | 18,945,398 | 3,827 | 95.84 |  |  |
| Lid | 15,144,053 | 14,913,623 | 3,013 | 96.08 |  |  |
| Pooled | 270,564,265 | 262,352,661 |  |  |  |  |

**Table S2**. Alignment summary of the individual reads to the reference transcriptome.

| Description | Leaf base | Tendril | Digestive zone | Waxy zone | Lid |
| --- | --- | --- | --- | --- | --- |
| *Sample 1* | | | | | |
| Raw paired-end reads passing Illumina's quality filter | 39,148,056 | 35,261,164 | 47,190,247 | 30,171,870 | 28,288,959 |
| No. of pre-processed paired-end reads | 14,845,898 | 33,676,794 | 41,585,967 | 27,507,082 | 26,004,134 |
| No. of reads aligned | 13,640,411 | 31,949,174 | 40,026,493 | 25,644,852 | 25,083,588 |
| Alignment percentage | 91.88% | 94.87% | 96.25% | 93.23% | 96.46% |
| No. of transcripts with FPKM ≥ 1.0 | 33,601 | 72,076 | 37,174 | 50,622 | 39,062 |
| *Sample 2* | | | | | |
| Raw paired-end reads passing Illumina's quality filter | 13,227,120 | 20,367,239 | 14,838,985 | 18,945,398 | 14,913,623 |
| No. of pre-processed paired-end reads | 8,666,372 | 19,979,561 | 11,241,050 | 13,116,687 | 14,035,176 |
| No. of reads aligned | 8,226,120 | 19,138,421 | 10,768,926 | 12,523,812 | 13,375,522 |
| Alignment percentage | 94.92% | 95.79% | 95.80% | 95.48% | 95.30% |
| No. of transcripts with FPKM≥ 1.0 | 30,186 | 87,277 | 27,767 | 37,656 | 38,942 |
| *Mean coefficient of variation** | | | | | |
| Assembled transcripts  Assembled transcripts showing expression in each tissue | 0.38  1.09 | 0.66  0.95 | 0.42  1.07 | 0.66  1.14 | 0.45  1.06 |

*The coefficient of variation (CV) of the FPKM values generated for each transcript in each part/zone of the *N. khasiana* leaf was calculated in Microsoft Excel using the formula CV=standard deviation/mean. The mean CV was then calculated across all transcripts in each sample. We generated two mean CVs: one in which all transcripts are considered (assembled transcripts) and another in which only assembled transcripts showing expression in each tissue are included in the calculations. The mean CV was calculated to provide a measure of the relative variability between the two datasets. No selection of stable or consistently expressed transcripts was performed.

**Table S3**. List of qPCR primers used for the validation of RNA-seq derived transcript expression pattern.

| Sl. No. | Gene (Abbreviated) | Gene (Expanded form) | Primer sequence |
| --- | --- | --- | --- |
| *Endogenous genes for normalization* | | | |
| 1 | *NkActin-1-Forward* | *Nepenthes khasiana* Actin (1) | 5'- TCTCAACCCCAAAGCAAACAG -3' |
|  | *NkActin-1-Reverse* |  | 5'- GCAGGGACATTGAAGGTCTCA -3' |
| 2 | *NkActin-2-Forward* | *Nepenthes khasiana* Actin (2) | 5'- TCCCTGGCATTGCTGACA -3' |
|  | *NkActin-2-Reverse* |  | 5'- GCGGAGCAACCACCTTGAT -3' |
| 3 | *NkELF-Forward* | *Nepenthes khasiana* Elongation Factor | 5'- CAAGTCACCCAACAAGCACAA -3' |
|  | *NkELF-Reverse* |  | 5'- CAGCAAGACCATCCTCCATAGG -3' |
| 4 | *NkGAPC2-Forward* | *Nepenthes khasiana* Glyceraldehyde-3-phosphate dehydrogenase | 5'- GTGGTGCTAAGAAGGTCGTCATC -3' |
|  | *NkGAPC2-Reverse* |  | 5'- TCTCATTAACACCAACAACGAACA -3' |
| 5 | *NkUBQ-Forward* | *Nepenthes khasiana* Ubiquitin | 5'- CGAAGATTCAAGACAAGGAGGGTAT -3' |
|  | *NkUBQ-Reverse* |  | 5'- CCATCCTCCAACTGCTTACCA -3' |
| *Genes involved in prey digestion and plant defence* | | | |
| 6 | *NkPAP-Forward* | *Nepenthes khasiana* Purple Acid Phosphatase | 5'- GGCCTCGCCACAACGTATAT -3' |
|  | *NkPAP-Reverse* |  | 5'- CATGGCCAAAGCTTGCTTCT -3' |
| 7 | *NkAP-Forward* | *Nepenthes khasiana* Aspartic protease | 5'- TTGACACAGGCAGTGCTAATCTCT -3' |
|  | *NkAP-Reverse* |  | 5'- TGGAAAAAACAAGGGACTGAGAA -3' |
| 8 | *NkCHITIV-Forward* | *Nepenthes khasiana* Chitinase IV | 5'- CTTCGGAGCCACCATTCG -3' |
|  | *NkCHITIV-Reverse* |  | 5'- ACGATCATCGACGGCAGAA -3' |
| 9 | *NkNEPI-Forward* | *Nepenthes khasiana* Nepenthesin I | 5'- GATTTGGAGTTGCCCAGTGTG -3' |
|  | *NkNEPI-Reverse* |  | 5'- CGGTGTCGTAAACGACTAG -3' |
| 10 | *NkNEPII-Forward* | *Nepenthes khasiana* Nepenthesin II | 5'- ATATCTGTCCGGTGAGCAGCTG -3' |
|  | *NkNEPII-Reverse* |  | 5'- GAGTGAAACCCAGTAGTTGG -3' |
| 11 | *NkSCPL-Forward* | *Nepenthes khasiana* Serine carboxypeptidase | 5'- AGTCGCCGGGTACACACAAG -3' |
|  | *NkSCPL-Reverse* |  | 5'- TGTCCAGACCCCTTAACAGTAAGAA -3' |
| 12 | *NkRNase-Forward* | *Nepenthes khasiana* S-like Ribonuclease | 5'- CGTGCAACACCGACGAATAC -3' |
|  | *NkRNase-Reverse* |  | 5'- AGAATGGCCGACACAAATGTAA -3' |
| 13 | *NkPKS-Forward* | *Nepenthes khasiana* type III polyketide synthase | 5'- ATGAGGAAGAGGTCTATGGAGGAA -3' |
|  | *NkPKS-Reverse* |  | 5'- CAAACAAGACACCCCATTCAAA -3' |
| *Stomatal development* | | | |
| 14 | *NkYDA-Forward* | *Nepenthes khasiana YODA* | 5'- CCTTGGATGCACTGTATTGGAA -3' |
|  | *NkYDA-Reverse* |  | 5'- TGGCAGCAACCCCTTCATA -3' |
| 15 | *NkMKK5-Forward* | *Nepenthes khasiana* Mitogen-activated protein kinase (MAPK) kinase | 5'- CCCTTCAACACCAATCCTTCTG -3' |
|  | *NkMKK5-Reverse* |  | 5'- GCTTCCGCTACCGATTCG -3' |
| 16 | *NkMPK3-Forward* | *Nepenthes khasiana* Mitogen-activated protein (MAP) kinase | 5'- TTGATGGACCGAAAGCCTCTA -3' |
|  | *NkMPK3-Reverse* |  | 5'- CAATCAGCTCCATAAGCAATCG -3' |
| 17 | *NkSCRM1-Forward* | *Nepenthes khasiana* SCREAM | 5'- CCTGTCGTGTAAAGGAGGAGCTAT -3' |
|  | *NkSCRM1-Reverse* |  | 5'- GCCCTTACTTCAACCCTTGCT -3' |
| *Leaf polarity genes* | | | |
| 18 | *NkPHB-Forward* | *Nepenthes khasiana PHABULOSA* | 5'- GATGTCTTGATGTCCTGAGTGTGAT -3' |
|  | *NkPHB-Reverse* |  | 5'- TGTATCGCAATGTCCAAAAGTCA -3' |
| 19 | *NkREV-Forward* | *Nepenthes khasiana REVOLUTA* | 5'- TCCTACCACCTCTAACCCTGCTAA -3' |
|  | *NkREV-Reverse* |  | 5'- CATGGATGCCTTTGCACAGA -3' |
| 20 | *NkKAN1-Forward* | *Nepenthes khasiana KANADI 1* | 5'- AAGAGCCATTTACAGATGTATCGAACT -3' |
|  | *NkKAN1-Reverse* |  | 5'- TTCCTCTACGTATCCATCAAATTGTC -3' |
| 21 | *NkKAN4-Forward* | *Nepenthes khasiana KANADI 4* | 5'- CAGGGAAAGATATAAAGGCAATGG -3' |
|  | *NkKAN4-Reverse* |  | 5'- GCAACAGTCTCTCCTTCTCTTTCTTC -3' |
| 22 | *NkARF3-Forward* | *Nepenthes khasiana AUXIN RESPONSE FACTOR 3* | 5'- CATCTGCTATATTCCCAGGTCAAGT -3' |
|  | *NkARF3-Reverse* |  | 5'- GGCCAAGACTCCTCAAAAACC -3' |
| 23 | *NkARF4-Forward* | *Nepenthes khasiana AUXIN RESPONSE FACTOR 4* | 5'- AGTGGAGGTTTCGACACATTTATAGAG -3' |
|  | *NkARF4-Reverse* |  | 5'- GCATCCCCCGAAACAAGA -3' |
| 24 | *NkAS1-Forward* | *Nepenthes khasiana ASYMMETRIC LEAVES1* | 5'- AAGCACGGCAACAAATGGA -3' |
|  | *NkAS1-Reverse* |  | 5'- ACCACTTGCCCAGCCTCTT -3' |
| 25 | *NkAS2-Forward* | *Nepenthes khasiana ASYMMETRIC LEAVES2* | 5'- GCGGCGGGTTTAATCAGAT -3' |
|  | *NkAS2-Reverse* |  | 5'- TTGGATTAGGTAAGGGTCATTGAAG -3' |
| 26 | *NkYAB1- Forward* | *Nepenthes khasiana YABBY1* | 5'- AAGACTGTGACGGTGCGATGT -3' |
|  | *NkYAB1- Reverse* |  | 5'- GGGCGGCAGGAGCAA -3' |
| 27 | *NkYAB5- Forward* | *Nepenthes khasiana YABBY5* | 5'- AAGTTTATAAAGGAGGAGATCCAGAGAA -3' |
|  | *NkYAB5- Reverse* |  | 5'- GGCCAAAATGAATATCAGGGAAA -3' |
| 28 | *NkAGO1-Forward* | *Nepenthes khasiana ARGONAUTE 1* | 5'- TGTCAGTGACCGACCTACGATTA -3' |
|  | *NkAGO1-Reverse* |  | 5'- GGGCTTGAATCTTCTCCAGGAT -3' |
| 29 | *NkAGO10-Forward* | *Nepenthes khasiana ARGONAUTE 10* | 5'- TCAGTCGCTAACCAACAACCTTT -3' |
|  | *NkAGO10-Reverse* |  | 5'- CCGGTGGAGCGACAGAGA -3' |
| *Randomly selected genes for additional validation using qPCR* | | | |
| 30 | *NkCYTC-Forward* | *Nepenthes khasiana* Cytochrome c biogenesis protein | 5'- AAAAGAAGCCGCTATGGTGAAA -3' |
|  | *NkCYTC-Reverse* |  | 5'- TCGAACCGAGATGCTCTAGCA -3' |
| 31 | *NkPRP1-Forward* | *Nepenthes khasiana* Pathogen-related protein 1 | 5'- GAGAACCTAGCATGGGCATCA -3' |
|  | *NkPRP1- Reverse* |  | 5'- GCTTCTCGTTGACCCACAAGTT -3' |
| 32 | *NkLAC4-Forward* | *Nepenthes khasiana* Laccase-4-like | 5'- CCAGGAAGTGGTGGTCGTTT -3' |
|  | *NkLAC4- Reverse* |  | 5'- ATCGGAGACGTTCGGTGCTA -3' |
| 33 | *NkPRP-Forward* | *Nepenthes khasiana* Pathogenesis-related protein | 5'- CAACCACCGAGGGAGACGTA -3' |
|  | *NkPRP- Reverse* |  | 5'- GTGGCCAGCTTGCAGATACTG -3' |
| 34 | *NkCAB-Forward* | *Nepenthes khasiana* Chlorophyll a-b binding protein 8 | 5'- AAACCCGTATCGAAAAAAAGCA -3' |
|  | *NkCAB- Reverse* |  | 5'- TCTATGGGCTCTCAGATATTTGTAATTG -3' |
| 35 | *NkD1-Forward* | *Nepenthes khasiana* photosystem II protein D1 | 5'- TGGATTGCTGTTGCGTATTCA- 3' |
|  | *NkD1- Reverse* |  | 5'- GAAAAGCTTCCTTGACCAATTGG- 3' |
| 36 | *NkPXD-Forward* | *Nepenthes khasiana* Putative peroxidase | 5'- ACAACACCGCCCCTCTCA -3' |
|  | *NkPXD- Reverse* |  | 5'- ACCAGGAGGTTGCCGTAGTAGA -3' |
| 37 | *NkNAC-Forward* | *Nepenthes khasiana* NAC domain-containing protein | 5'- CAGCCTTCACCGCCTTGT -3' |
|  | *NkNAC- Reverse* |  | 5'- ATGAATCTATTGGTGGCAGTGATG -3' |

**References**

1. Salisbury EJ. On the causes and ecological significance on stomatal frequency with special reference to woodland flora. Phil. Trans. Roy. Soc. Lon. Ser. B 1927;216:1‒65.
2. Nadeau JA, Sack FD. Control of Stomatal Distribution on the *Arabidopsis* Leaf Surface. Science 2002;296:1697‒1700.
3. Yang M, Sack FD. The *too many mouths* and *four lips* mutations affect stomatal production in Arabidopsis. Plant Cell 1995;7:2227‒2239.
4. Berger D, Altmann T. A subtilisin-like serine protease involved in the regulation of stomatal density and distribution in *Arabidopsis thaliana*. Genes Dev. 2000;14:1119‒1131.
5. Shpak ED, McAbee JM, Pillitteri LJ, Torii KU. Stomatal patterning and differentiation by synergistic interactions of receptor kinases. Science 2005;309:290‒293.
6. Wang H, Ngwenyama N, Liu Y, Walker JC, Zhang S (2007) Stomatal Development and Patterning Are Regulated by Environmentally Responsive Mitogen-Activated Protein Kinases in Arabidopsis. Plant Cell 2007;19:63‒73.
7. Bergmann DC, Lukowitz W, Somerville CR (2004) Stomatal Development and Pattern Controlled by a MAPKK Kinase. Science 2004;304:1494‒1497.
8. Hara K, Yokoo T, Kajita R, Onishi T, Yahata S, Peterson KM, et al. Torii KU, Kakimoto T. Epidermal cell density is autoregulated via a secretory peptide, EPIDERMAL PATTERNING FACTOR 2 in Arabidopsis Leaves. Plant Cell Physiol. 2009;50:1019‒1031.
9. Hara K, Kajita R, Torii KU, Bergmann DC, Kakimoto T The secretory peptide gene *EPF1* enforces the stomatal one-cell-spacing rule. Genes Dev. 2009;21:1720‒1725.
10. Sugano SS, Shimada T, Imai Y, Okawa K, Tamai A, Mori M, et al. Stomagen positively regulates stomatal density in Arabidopsis. Nature 2010;463:241‒244.
11. Lai LB, Nadeau JA, Lucas J, Lee E, Nakagawa T, Zhao L, et al. The *Arabidopsis* R2R3 MYB proteins FOUR LIPS and MYB88 restrict divisions late in the stomatal cell lineage. Plant Cell 2005;17:2754‒2767.
12. Pillitteri L, Sloan DB, Bogenschutz NL, Torii KU. Termination of asymmetric cell division and differentiation of stomata. Nature 2006;445:501‒505.
13. Ohashi-Ito K, Bergmann DC. *Arabidopsis* FAMA controls the final proliferation/differentiation switch during stomatal development. Plant Cell 2006;18:2493‒2505.
14. MacAlister CA, Ohashi-Ito K, Bergmann DC. Transcription factor control of asymmetric cell divisions that establish the stomatal lineage. Nature 2007;445:537‒540.
15. Kanaoka MM, Pillitteri LJ, Fujii H, Yoshida Y, Bogenschutz NL, Takabayashi J, et al. *SCREAM/ICE1* and *SCREAM2* specify three cell-state transitional steps leading to *Arabidopsis* stomatal differentiation. Plant Cell 2008;20:1775‒1785.
16. Groll U Von, Berger D, Altmann T. The subtilisin-like serine protease SDD1 mediates cell-to-cell signaling during Arabidopsis stomatal development. Plant Cell 2002;14:1527‒1539.
